# Significant hot hand effect in the game of cricket

**DOI:** 10.1038/s41598-022-14980-7

**Published:** 2022-07-08

**Authors:** Sumit Kumar Ram, Shyam Nandan, Didier Sornette

**Affiliations:** 1 Connection Science, Massachusetts Institute of Technology, Cambridge, USA; 2 Department of Management, Technology and Economics, ETH Zürich, Scheuchzerstrasse 7, 8092 Zurich, Switzerland; 3 Swiss Seismological Service, ETH Zürich, Sonneggstrasse 5, 8092 Zurich, Switzerland; 4 Institute of Risk Analysis, Prediction and Management (Risks-X), Academy for Advanced Interdisciplinary Studies, Southern University of Science and Technology (SUSTech), Shenzhen, China

**Keywords:** Nonlinear phenomena, Statistical physics, Computational science

## Abstract

We investigate the predictability and persistence of individual and team performance (hot-hand effect) by analyzing the complete recorded history of international cricket. We introduce an original temporal representation of performance streaks, which is suitable to be modelled as a self-exciting point process. We confirm the presence of predictability and hot-hands across the individual performance and the absence of the same in team performance and game outcome. Thus, Cricket is a game of skill for individuals and a game of chance for the teams. Our study contributes to recent historiographical debates concerning the presence of persistence in individual and collective productivity and success. The introduction of several metrics and methods can be useful to test and exploit clustering of performance in the study of human behavior and design of algorithms for predicting success.

## Introduction

The study of what bring success or failure in battles and wars, in politics, in business, in sports, even in our personal lives, has a very long history, being part of the DNA of human evolution that has tended to promote the genes of the “successful ones”^1^. The ‘science of success’ has received a boost in recent years with the growing availability of large datasets describing individual’s careers from which much can be learned and importantly predicted^2–10^. The increasing shift towards collaborative and team-based effort (performance) in recent decades has made it more important to quantify and predict teamwork^11–15^. However, the translation of the predictability in individual performance to team performance is still missing.

**Figure 1:**
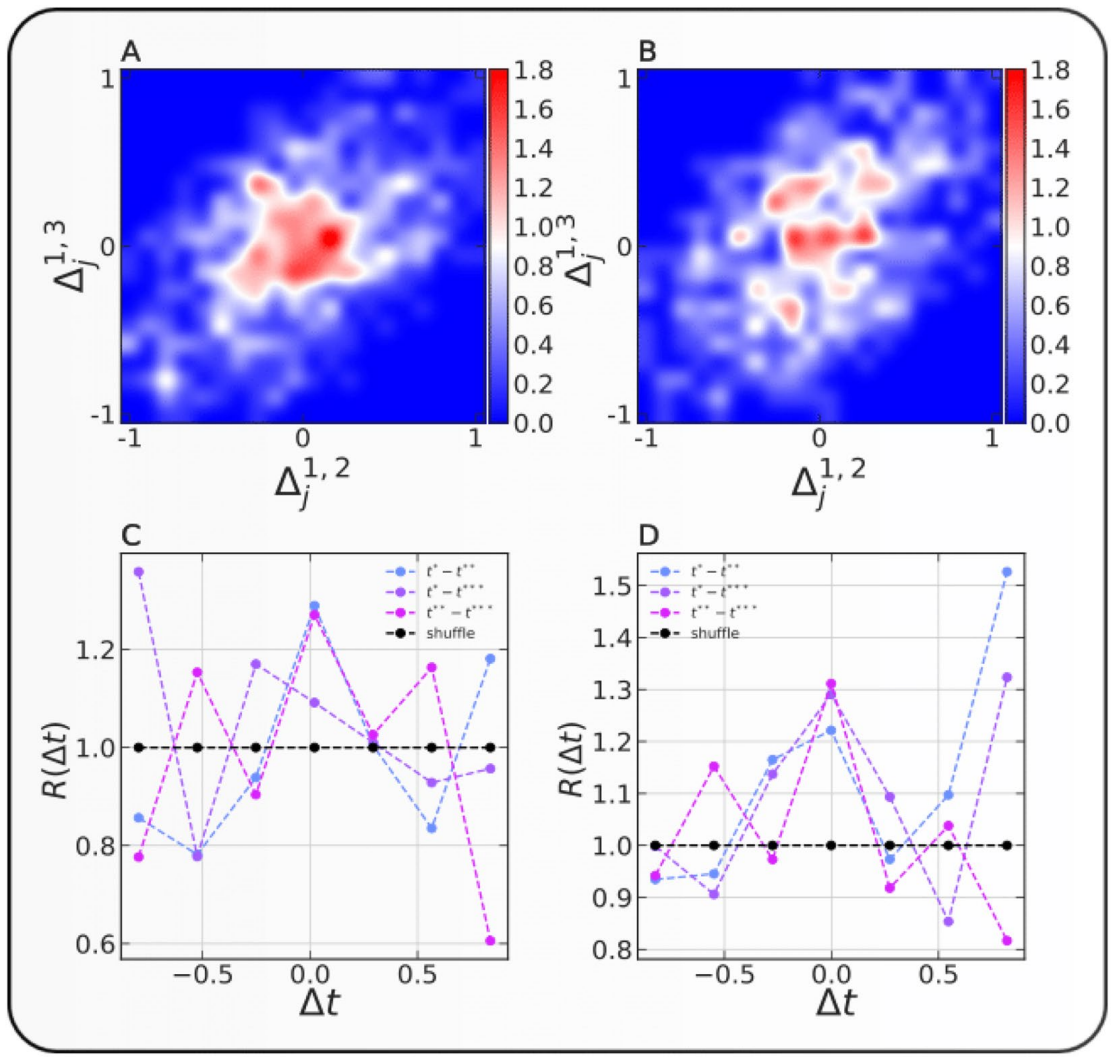
Joint probability distribution and $Q( {\Delta_{j}^{1,2} ,\Delta_{j}^{1,3} } )$ and $R( {\Delta t} )$. (**A**,**B**) show the joint distribution of the relative difference of the indices of second best from the best (defined by Eq. (2)  in Subsection "Distributions of temporal locations of best performances"), plotted against the third best from the best performances. (**C**,**D**) show $R( {\Delta t} )$ defined by Eq. (3) for $\Delta t = \Delta_{j}^{1,2} ,\Delta_{j}^{1,3} ,\Delta_{j}^{2,3}$. (**A**,**C**) correspond to the batting performances in ODI cricket and (**B**,**D**) correspond to the performances in Test cricket.

In this study, we develop novel statistical tools to uncover the temporal features that are characteristic of a set of performances. We explore the complete history of International cricket^16,17^ to quantify individual and team performances. We study the sequence of consecutive performances of each player and team. By investigating the scores of individual players against the index of the games within the career, we note that success breeds success in individual career (also supported by ARIMA model in SM). We further document that the best performances in a given player’s career are clustered in time (see Fig. 1), contrary to previous findings^18,19^. However, we cannot say the same for teams. We uncover the presence of hot hands in individual careers in both formats of the game but the absence of the same in team performances. Our proposed Hawkes model applied to the performance time not only outperforms the traditional techniques like ARIMA and autocorrelation measures^20,21^ (see SM) but is successful in capturing the ingredients of self-excitation in the patterns of consecutive superior performances. These findings raise intriguing questions regarding the nature and extent of predictability of one’s success and team success in a team game. This is particularly interesting, since these findings not only refute the well-established narratives of the absence of hot hands in team games^18,19,22,23^ where performances are usually driven by stochastic events. Our findings suggest that the hot hand effect is not just a psychological bias^18,19^. A part of results corroborate previous works on hot-hands^8,9,24–26^. One of the possible explanations for the observation of such a peculiar behavior in the game of cricket may be the relatively larger importance of skill in the outcomes of a player’s game and luck in the outcomes of a teams’ game^10,27^.

The rest of the article is structured as follows. In section “Literature review”, we present a short literature review to motivate our study and put it in the right context. Section "Methods" includes three subsections Dataset, Distributions of temporal locations of best performances and Clustering point process representation. Subsection “Dataset” describes the dataset that has been used in the study and the data acquisition methodology. Subsection “Distributions of temporal locations of best performances” summarizes the empirical observations. Subsection “Clustering point process representation” presents our proposed clustering point process representation in the form of a self-excited point process model to quantify and predict the hot hands in the sequences of performances. Section “Results and discussion” presents our main results. We conclude the results of the study in Section “Conclusion”.

### Literature review

A much-debated question is whether or not a string of successes of an individual or entity is more likely to cause continued success. When present, this is called The Hot Hand effect. When absent, the belief in it is called the hot-hand fallacy, whereas the belief in the opposite, i.e., success is less likely after a streak of success is called Gambler’s fallacy^28^. The question of whether the Hot Hand effect genuinely exists is important, as its positive answer has far-reaching consequences in several research fields, including finance and econometrics^10,26,29–31^, psychology^18,19,32,33^ and sociology^2,8,9,34,35^. The debate on the “Hot Hand fallacy” vs. the “Gambler’s Fallacy” revolves around the deeper question: ‘to what extent, human beings are capable of dealing with inherent systemic stochasticity’^10,27^. In their seminal paper, Gilovich et al. refute the validity of “the hot hand” and “streak shooting” in the game of basketball^18^. Their analyses of the shooting records of the Philadelphia 76ers, Boston Celtics, and a controlled shooting experiment with the men and women of Cornell’s varsity teams provided no evidence for a positive correlation between the outcomes of successive shots. They further showed that the belief in the hot hand and the detection of streaks in random sequences is nothing but an expression of the general misconception of chance^18^, according to which even short random sequences are thought to be highly representative of their generating process. There has been very strong support for this reasoning in the literature, especially in the field of finance and economics^23,30–32,36^. These studies support the idea that the hot-hand effect is a fallacy, stating that the hot hand does not exist and is nothing but a psychological bias based on the “law of small numbers”. Moreover, these studies warn that this fallacy may often lead people to take costly and risky decisions.

On the other side of the debate, Miller and Sanjurjo^24^ have recently challenged the original findings in^18^, with contrasting conclusions revealing significant evidence for streak shooting. Miller and Sanjurjo showed that the method used in^18^ introduced a sampling bias because they start counting after a series of hits/misses. They further showed that the method of^18^ is biased towards more misses, thus claiming that an equal rate of hits to misses after a streak presented in^18^ is, in fact, a sign of a hot hand. Csapo and Raab^37^ found evidence for the “hot hand” in that making the first free throw is associated with a significantly higher probability of making the second free throw. The debate about successful streaks has gained fresh prominence in many other fields, with many arguing for the presence of such streaks in large scale data sets of scientific careers, artistic career and acting careers^8,9,33,38,39^.

From the point of view of sport psychology, a belief built out of random sequential events can have positive effects on behavior. Athletes believe in the hot hand in volleyball and that streaks do exist for half of the players. Coaches can detect players' performance variability and use it to make strategic decisions, and playmakers are also sensitive to streaks and tend to use it "adaptively," which results in more hits for a team^40^. The belief in hot streaks can provide valid cues to decide who to give shots to, and this behavior is supported by the fallacious belief in dependency^41^. This is further validated in^42^, which analyzed the sequential choices made by expert athletes and found that they were sensitive to base rates and adapted their decision strategies accordingly. Additionally, defensive pressure and shot difficulty increase during hot streaks, so that defenders seem to behave according to the hot-hand belief and try to force hot players into more difficult shots^37^. Thus, even a single successful shot is enough to increase a player's likelihood of taking the next shot, and also to increase the average distance from which that shot is taken^43^. Arkes^44^ also found evidence for the “hot hand” in that making the first free throw is associated with a significantly higher probability of making the second free throw. However, the success of the next shot can be lower, while the coach is less likely to replace the player^43^. Additionally^37^, also found that shooting percentages of presumably hot players do not increase and that shooting performance is not related to streakiness, so that the defenders' hot-hand behavior cannot be considered ecologically rational.

The above debates revolve around the investigation of presence or absence of the hot-hand effect in individual performances. However, they fail to show how these effects can be exploited for better prediction or how the aggregated individual performances drive the evolution of team performance. In this study, we present a novel methodology to better understand and predict individual and team performances. We derive our methodology from the self-excited conditional Hawkes point process^45^, which has been applied in a variety of fields particularly the description of social diffusion processes^46–48^, financial systems^49–51^, and seismological predictions^52–54^. To the best of our knowledge, this is the first use of Hawkes processes in the domain of ‘science of success’. We apply our methodology for studying the presence (or absence) of the hot hand effect within the performance sequences in individual performance in the game of cricket. Our methodology would be useful in predicting and quantifying hot-hand effect in performance sequences in many other domains.

## Methods

### Dataset

**Figure 2:**
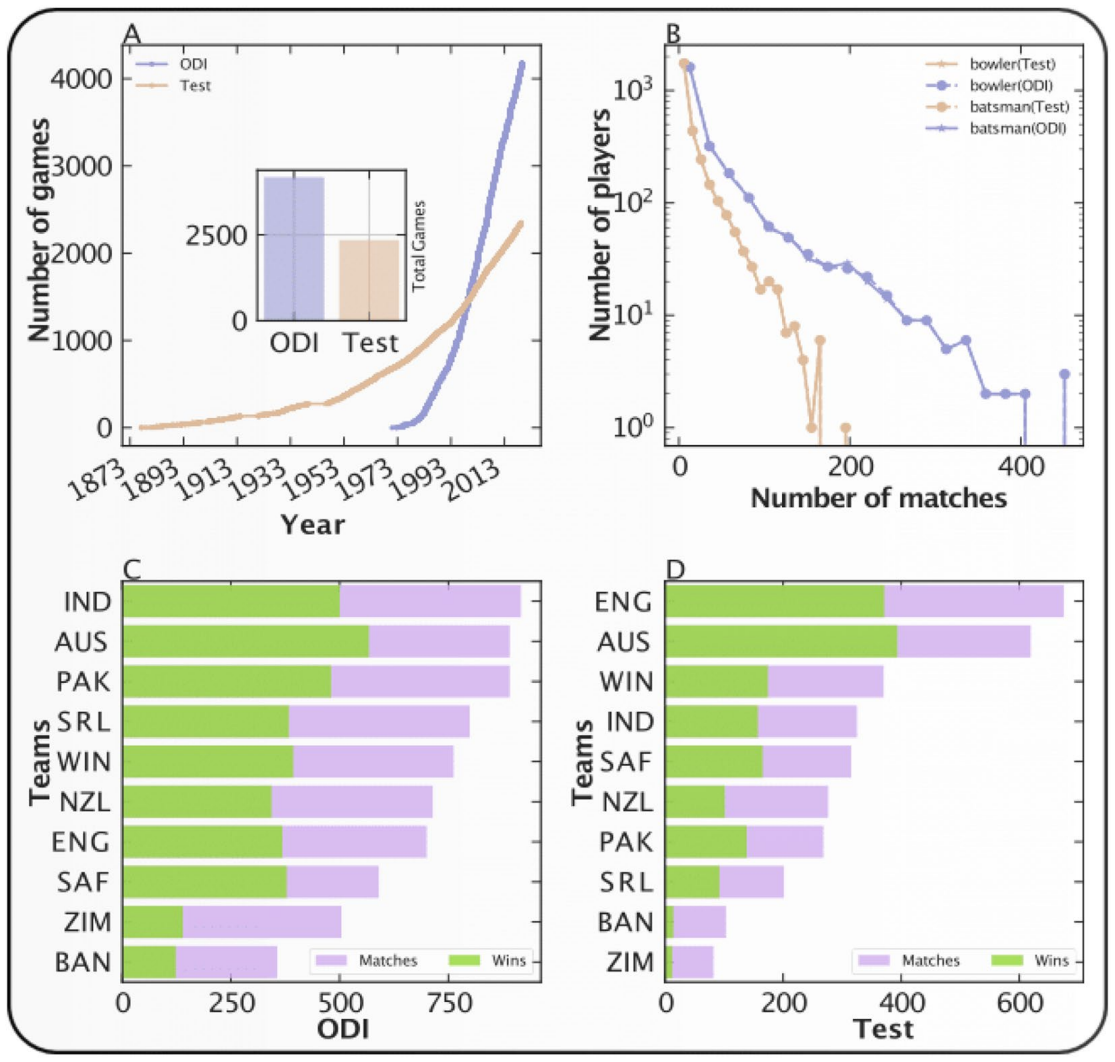
Visual representation of the database. (**A**) The time evolution of the total number of games in ODI and Test format. The inset figure represents the total number of games in each format. The brown color represents the Test cricket and the purple color represents the ODI cricket. (**B**) The joint distribution of the total number of players against the total number of games played by each player. The four symbols shown in the inset can be paired since the number of batsmen and of bowlers coincide by construction of the game. The total number of ODI games played and the total number of games won by each country is presented in (**C**). The same statistics for Test cricket is presented in (**D**). The purple color represents the total games and the green color represents the total wins.

The dataset we use in this study includes 4178 One Day International (ODI) games starting from January 5, 1971, till July 1, 2019 (48 years) and 2351 international Test games spanning March 1877 to March 2019 (142 years) (see SM for data acquisition and preparation). We record 51,699 batting performances of 2959 Test batsmen and 51,088 bowling performances of 2874 Test bowlers, 90,166 batting performances of 2500 ODI batsmen and 90,754 bowling performances of 2505 ODI bowlers (in total 283,707 records) (see Fig. 2). The dataset further contains the information about the performance of the teams and the outcomes of the games. To have meaningful calibration results, we only analyze the performances of those batsmen who have played at least 30 games (see goodness of fit in SM).

### Distributions of temporal locations of best performances

To study the self-excited nature of the scores in an individual’s career, we investigate the relative positions of the best three performances in each player’s career. We first order the games within one's career according to calendar time. We define the index (t) of any game by the rank of this game within this ordered sequence. We denote $t_{j}^{*}$ the index of the best performance in player *j*’s career, i.e.,

1 $$\begin{array}{*{20}c} { t_{j}^{*} = argmax_{t} S_{j} \left( t \right)} \\ \end{array}$$

Where, $${S_{\mathrm {j}} \left( \text{t} \right)}$$ is the performance of the player j at $$ {\mathrm{t}}^{\mathrm{th}} $$ attempt. We also define $$t_{j}^{**} , t_{j}^{***}$$ as the indices of the second, third best performance, and $$\tau_{j}$$ as the length of an individual’s career. We then calculate the relative difference of indices of the three best performances as

2 $$\Delta_{j}^{1,2} = \frac{{t_{j}^{*} - t_{j}^{**} }}{{\tau_{j} }},\Delta_{j}^{1,3} = \frac{{t_{j}^{*} - t_{j}^{***} }}{{\tau_{j} }},\Delta_{j}^{2,3} = \frac{{t_{j}^{**} - t_{j}^{***} }}{{\tau_{j} }}$$

for all players in our dataset and define the marginal probability density functions $P( {\Delta_{j}^{1,2} } ), P( {\Delta_{j}^{1,3} } ), P( {\Delta_{j}^{2,3} } )$ and the joint probability distribution $Q( {\Delta_{j}^{1,2} ,\Delta_{j}^{1,3} } )$. As a control, we shuffle the indices of the performances within the individual’s career and reevaluate these quantities. The primed quantities correspond to the shuffled career, i.e., $t_{j}^{\prime *}$ corresponds to the index of the best performance within the randomly reshuffled player *j’s* career, and so on. We define the marginal probability density functions $P( {\Delta_{j}^{\prime 1,2} } ), P( {\Delta_{j}^{\prime 1,3} } ), P( {\Delta_{j}^{\prime 2,3} } ),$ which are the distributions of the shuffled versions $\Delta_{j}^{\prime 1,2} \,{\text{of}}\,\Delta_{j}^{1,2} ,\Delta_{j}^{1,3} \,{\text{of}}\,\Delta_{j}^{\prime 1,3}$ and $\Delta_{j}^{\prime 2,3} \,{\text{of}}\,\Delta_{j}^{2,3}$. We define the ratios $R( {\Delta t} )$ of these marginal probabilities to quantify the temporal colocation of the best performances in an individual career

3 $$\begin{array}{*{20}c} {R\left( {\Delta t} \right) = \frac{{P\left( {\Delta t} \right)}}{{P^{\prime } \left( {\Delta t^{\prime } } \right)}},\quad {\text{where}}\quad \Delta t = \Delta_{j}^{1,2} ,\Delta_{j}^{1,3} \,{\text{or}}\,\Delta_{j}^{2,3} .} \\ \end{array}$$

Figure 1 presents the joint probability distribution of relative difference of indices of best and second-best against the best and third best $( {Q( {\Delta_{j}^{1,2} ,\Delta_{j}^{1,3} } )} )$ (top panels) defined by Eq. (2), for *ODI* and *Test* formats over all individuals’ careers. We observe a concentration of high probability around the origin (0,0) in both formats of the game. This correlation is interesting since this characteristic is a feature of the self-excited process and is not expected in a pure memoryless Poissonian process. We further compare the joint probability distribution $( {Q( {\Delta_{j}^{1,2} ,\Delta_{j}^{1,3} } )} )$ with the corresponding reshuffled joint probability distribution $( {Q( {\Delta_{j}^{{{\prime }1,2}} ,\Delta_{j}^{{{\prime }1,3}} } )} )$ and present in figure S2. The *p* values from 2D Kolmogorov–Smirnov two sample tests in figure S2 signifies the significant clustering around origin. This finding constitutes a first line of evidence for the existence of temporal clustering in the performances across players’ careers.

The bottom panels of Fig. 1 shows the ratio $R( {\Delta t} )$ (Eq. (3)), which compares the marginal probability distribution of the relative difference of the indices in the real careers against the indices obtained from shuffled careers. The distinctive peak around 0 in the plots provides additional support for clustering of performance within careers. $R( {\Delta t} )$ is approximately symmetric around the origin, indicating that the highest performances are equally likely to arrive before or after the second highest and third-highest scores. This pattern is expected from a self-excited process with approximately equal propensity for performance persistence among the best performance streaks^55,56^. This was shown in the context of earthquake time and space clustering. Here, we can think of the highest performance as equivalent to the main shock in a seismic sequence. Then, the main shock can be shown to be triggered by large events that occur before it (“foreshocks”) and the main shock itself triggers large events (“aftershocks”)^55,56^.

### Clustering point process representation

#### Definition of the “performance time”

We call $S_{j} ( t )$ the performance (see SM for more details about the game of cricket) of the player $j$ at his *t*th attempt within his career. We define the subordinate time process $H_{j} ( t )$ of the stochastic process $S_{j} ( t )$^57^ as

4 $$\begin{array}{*{20}c} {H_{j} \left( t \right) = \mathop \sum \limits_{{t_{i} = 1}}^{t} \frac{1}{{S_{j} \left( {t_{i} } \right)}}} \\ \end{array}$$

The $t \to H_{j} ( t )$ map represents a nonlinear transformation from the index $t$ onto an effective “performance time” of player $j$. $H_{j} ( t )$ denotes a transformed time-stamp at which the *t*th event takes place for player *j*. This defines a point process along “performance time” with the time stamps $\{ {H_{j} ( {t_{1} } ),H_{j} ( {t_{2} } ), \ldots ,H_{j} ( {t_{n} } ), \ldots } \}$. The intuition behind definition (4) is that a series of strong performance values $\{ {S_{j} ( {t_{i} } ), S_{j} ( {t_{i + 1} } ), \ldots } \}$ are transformed into closely clustered points in “performance time”. This allows us to analyze the relationship between performances in time using simple one-dimensional techniques. In other words, by transforming $S_{j} ( t )$, into $H_{j} ( t )$, we project the stochastic process described by the sequence $\{ {S_{j} ( t ),t = 1, \ldots } \}$ onto an one-dimensional point process with time stamps $\{ {H_{j} ( {t_{1} } ), H_{j} ( {t_{2} } ), \ldots , H_{j} ( {t_{n} } ), \ldots } \}$. By construction, the $t \to H_{j} ( t )$ transformation preserves the self-excited component of performance scores described by the stochastic process $\{ {S_{j} ( t )} \}$ and amplifies it by the magnitude of the performance values.

**Figure 3:**
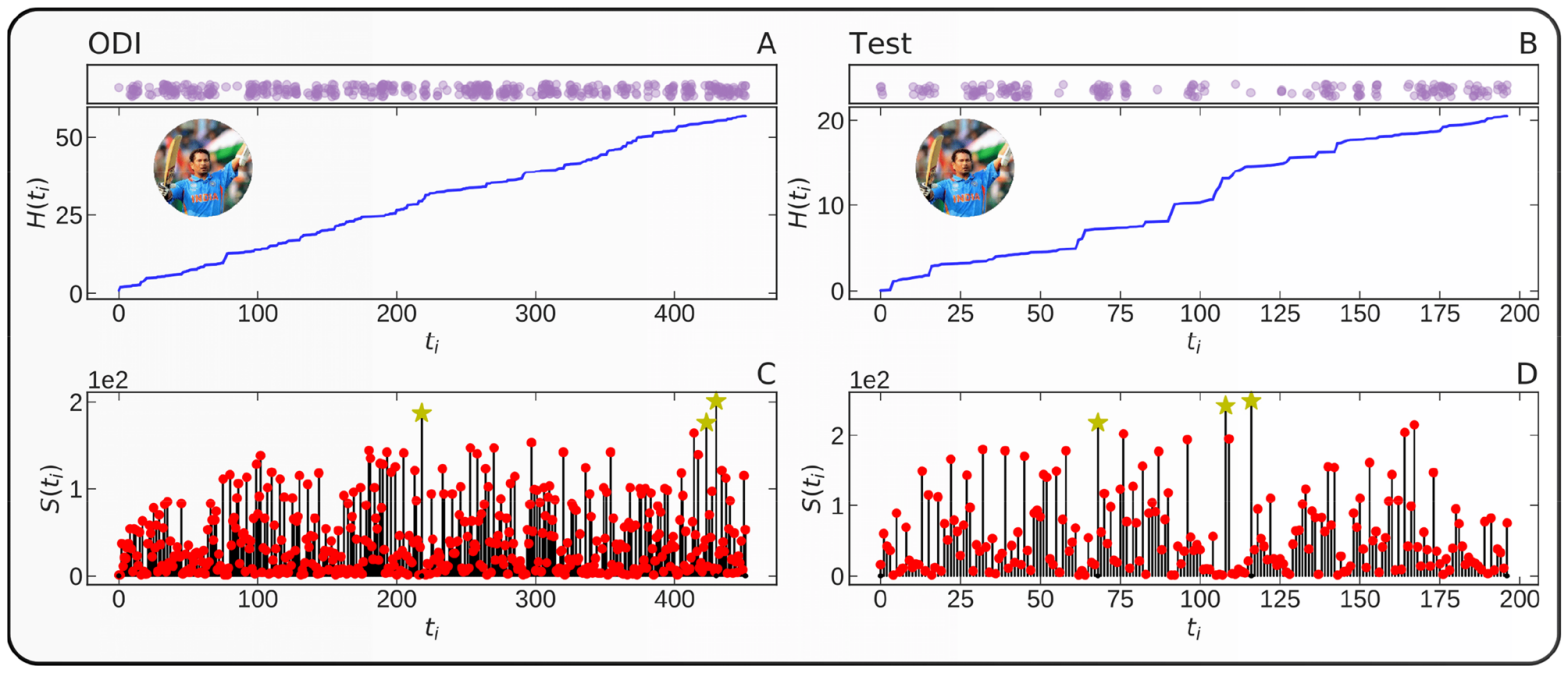
Sequence of performances in the individual career of Sachin Tendulkar. (**A**) Performance time $H\left( t \right)$ as a function of $t$, as defined in Eq. (4), for the highest performer in ODI cricket. (**B**) Performance time H(t) for the highest run scorer in Test cricket. (**C**) The performance score $S\left( t \right)$ of the player in ODI corresponding to panel (**A**). (**D**) The performance score $S\left( t \right)$ of the player in Test cricket corresponding to panel (**B**). The large yellow stars represent the top 3 performances. The top insets in (**A**,**B**) give the point process representation of $H\left( t \right)$, in which each dot corresponds to an instant of time along the $H\left( t \right)$ time axis. We have added noise along the y-direction for better visualization.

Figure 3 presents the example of the career of Sachin Tendulkar, who has the highest sum of performances in both formats of the game. Top panels show the performance time $H( t )$ as a function of *t*, t is the index of the *t*th attempt, as defined in Eq. (4), for two international cricketing formats, ODI and Test. Bottom panels show the scores $S_{j} ( t )$ as a function of $t$, for the two international cricketing formats, ODI and Test. The presence of local temporal clustering around the high and low performances is clearly visible in both representations of $H( t )$ and $S( t )$ for this player.

#### Hawkes point process along the “performance time”

The performance time $H_{j} \left( t \right)$ of player *j* defined by expression (4) allows us to introduce a point process by the performance times $\{ H_{j} \left( {t_{1} } \right),H_{j} \left( {t_{2} } \right), \ldots ,H_{j} \left( {t_{n} } \right), \ldots \}$ along the $H$ axis. In other words, we consider the “performance time” axis $H_{j} \left( t \right)$ and, along this new time axis, we identify “points” at the locations $\{ H_{j} \left( {t_{1} } \right),H_{j} \left( {t_{2} } \right), \ldots ,H_{j} \left( {t_{n} } \right), \ldots \}$. When player $j$ has a series of large scores, this is expressed as a cluster of closely spaced points along the $H$ axis as shown in Fig. 3.

Inspired by the analyses of^46,49,51^ using generalized non-homogeneous Poisson processes, we propose to model the clustering of the points along the *H* axis of each player by using the self-excited stochastic Hawkes point process model^45,50^, augmented by some necessary ingredients for constructing a prediction model^19^. In other words, we visualize the points for a given player *j* along the performance time axis $H_{j} \left( t \right)$ as being generated by a Hawkes model with intensity $\lambda \left( t \right)$ given by

5 $$\begin{array}{*{20}c} {\lambda \left( t \right) = \mu + \mathop \sum \limits_{{t_{i} < t}} \varphi \left( {t - t_{i} } \right)} \\ \end{array}$$

In expression (5), the first term μ in the right-hand-side is the background intensity, which quantifies the “intrinsic” performance level of a player, uninfluenced by his/her past performances. The second term describes how past points can trigger future points along the *H* axis. This is a convenient and elegant way to account for the possibility of a hot-hand effect, since each next point is function of the whole history, with a weight quantified by the memory or kernel function $\varphi \left( {t - t_{i} } \right) > 0$, which is decaying as a function of its argument (points further in the past have a weaker influence). Thus, the sum $\mathop \sum \nolimits_{{t_{i} < t}} \varphi \left( {t - t_{i} } \right)$ quantifies the influence of the history of past performances on a player’s present performance.

Depending on the problem, previous researchers have used different parametric forms for, e.g.^46,53,54^ use a power law kernel, whereas^58^ use an exponential kernel. In the present case, as there is no reason to favor any parametric form, we decide to use a non-parametric kernel function for φ^50,59^. Thus, shortly after a large performance amplitude, model (2) describes the possibility that the excess intensity of observing a similar performance is boosted and then decays to the baseline average performance level $\mu$ at long times.

The self-excited Hawkes conditional point process is one of the simplest models to account for how the past can influence the future, while keeping a very convenient linear dependence of the past onto the future. The most important parameter of the Hawkes model is its branching ratio defined by

6 $$\begin{array}{*{20}c} {n = \mathop \int \limits_{0}^{\infty } \varphi \left( t \right)dt.} \\ \end{array}$$

The branching ratio *n* is the average number of points (or events) of first generation triggered by a given point. It is also the fraction of points (events) that have been triggered by past events^60^. A value of n close to the critical value 1 thus qualifies a large level of triggering (strong hot hand effect) and endogeneity. Please see figure S4 for details about the used method.

We use the expectation maximization algorithm as described^50^ to calibrate the model.

## Results and discussion

### Hot individual hands

We partition the career of a player *j* into training set and validation set. We take the first 80% of the performances as the training set and the next 20% as the validation set. We transform the performance sequence in training and validation set to performance time representation (4) as discussed in “Methods” section. We calibrate the performance time in training set to determine background intensity $\mu$ and the memory kernel $\varphi$. We then use the calibrated $\mu$ and $\varphi$ to evaluate the prediction performance in validation set using the log-likelihood score and call the median value ${\mathcal{L}}_{j}^{model}$.

Similarly, we prepare a controlled set of log-likelihood estimation for the same player. Keeping the validation set unaltered, we shuffle the sequence of the performance in the training set *100* times and use this to train the model. We evaluate the trained model on the unaltered validation set to determine the corresponding median log-likelihood estimation ${\mathcal{L}}_{j}^{control}$. With the above constructions, we define the relative differences $\delta ( {{\mathcal{L}}_{j}^{model} ,{\mathcal{L}}_{j}^{control} } )$ by

7 $$\begin{array}{*{20}c} {\delta \left( {{\mathcal{L}}_{j}^{model} ,{\mathcal{L}}_{j}^{control} } \right) = \frac{{{\mathcal{L}}_{j}^{model} - {\mathcal{L}}_{j}^{control} }}{{{\mathcal{L}}_{j}^{control} }} } \\ \end{array}$$

Additionally, we estimate the branching ratios (see Eq. (6))^46,49,53^ of the performance time for all players over the duration of their entire career. For comparison, we construct null estimations by randomly shuffling the performance time times and reevaluating the 100 null branching ratios for each of the players.

**Figure 4:**
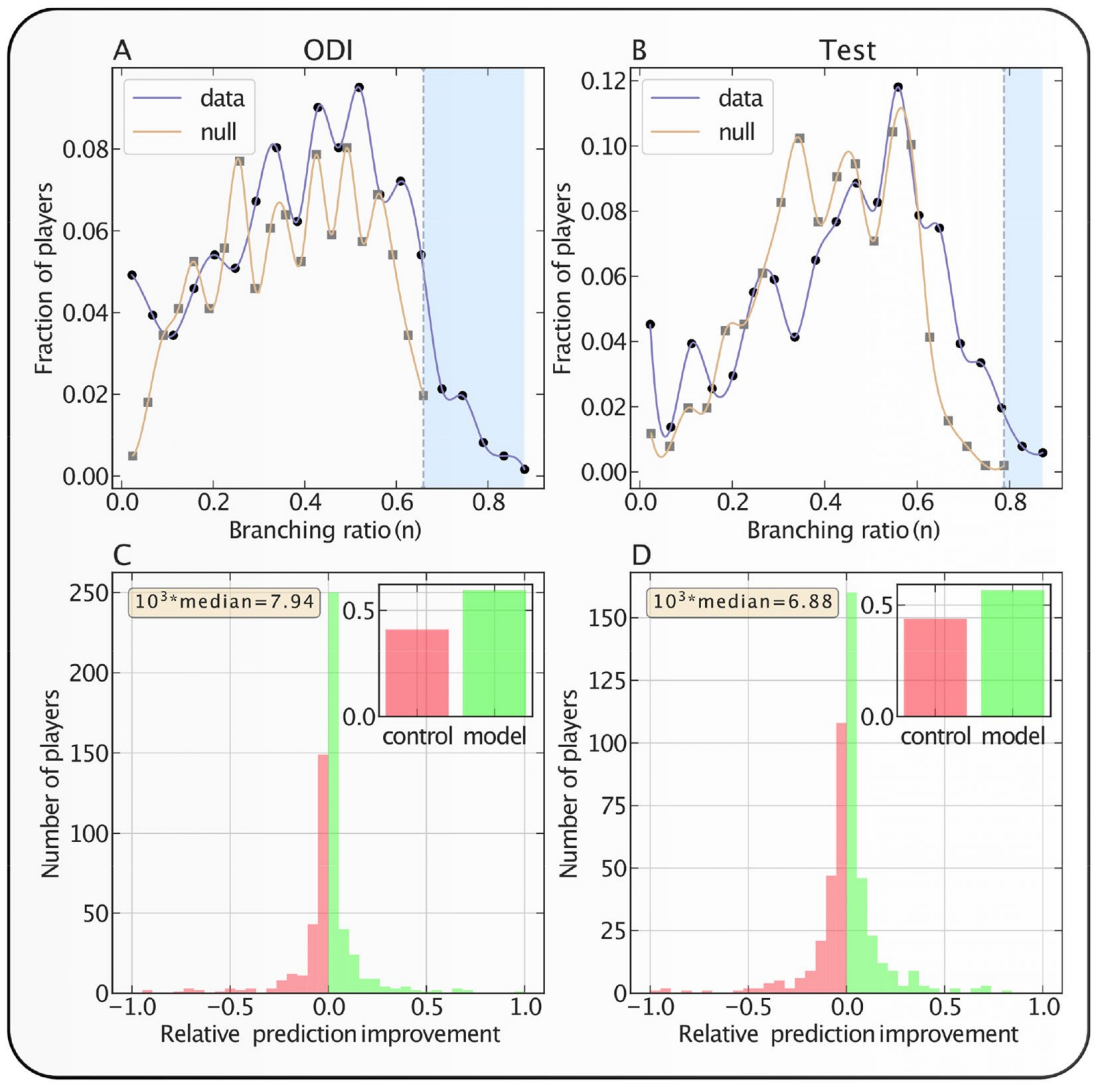
Analysis of clustering in the time series of performance time with the self-excited point process model. (**A**,**B**) represent the distribution of branching ratios over the set of players and of the branching ratios obtained from synthetic shuffled careers. (**A**) represents the distribution for ODI cricket and (**B**) is for Test cricket. Shaded regions in the plot represent the domain of branching ratios obtained from the real data that cannot be explained by the null models. (**C**,**D**) show the distribution of $\delta ( {{\mathcal{L}}_{j}^{model} ,{\mathcal{L}}_{j}^{control} })$ (see Eq. (7)). (**C**) represents the distribution for ODI cricket, and (**D**) is for the Test format. The fraction of the times models achieve a better log-likelihood score compared to the controls is colored green, otherwise the color is red. The insets show the fraction of controls and models outperforming their counterparts. In ODI games, the fraction of times models perform better than the controlst is: 0.62; for Test cricket, this fraction is 0.60.

The relative difference of log-likelihood prediction scores in Eq. (7) is shown in the bottom panels of Fig. 4, for both formats of the games. The insets present the fraction of time control performing better and the fraction of time the model performing better. The results show a significant improvement in prediction score in model experiments compared to the control experiments. We plot the distribution of the branching ratios obtained from the data and the null branching ratios and compare them in the top panels of Fig. 4. In the plots, the shaded region marks the fraction of players’ branching ratios that are never found in the null models. This behavior is robust against the number of simulated null models, i.e., the fraction of players’ branching ratios that are never found in the null model remains the same even if we consider 500 and 1000 null models.

We then compare the log-likelihood score from 100 control estimates with the log-likelihood score obtained from the data for each of the player. We evaluate the statistical significance of having a better log-likelihood score in the model experiments compared to the control experiments. We perform the Wilcoxon signed-rank test in each career to determine the statistical significance. Considering a confidence level of 0.05, we observe that, in 49.6% of Test careers and in 46.8% of the ODI careers, the log-likelihood prediction score in original sequences is significantly higher than the median log-likelihood prediction score in control experiments. This leads us to conclude that the probability of falsely accepting the null hypotheses—the control experiments perform equally good—is $< 10^{ - 6}$(using a binomial probability distribution with success rate 0.05 of false test result) for both the cases. This result is sufficient to support the predictive power of our model. Furthermore, our model performs better than the standard techniques like ARIMA and autocorrelation measures^20,21^ (please refer to the SM).

We then compare the branching ratios (see Eq. (6)) of the performance time obtained from data and null shuffling for each player to quantify the Hot-Hand effect. We perform the Wilcoxon signed-rank test to determine the statistical significance. We observe that in 56.8% of Test careers and in 53.7% of the ODI careers, the branching ratio of original performance time is significantly higher than the median branching ratio in null performance time (confidence level = 0.05). These results suggest a significant presence of Hot Hands in the players career, as the probability for the absence of Hot Hands is $< 10^{ - 6}$(using a binomial probability distribution with success rate 0.05 of false test result).

### Hot team hands

We repeat the above analysis to predict and quantify the team performances (sum of all individual performances in a game) (please see SM for more details). We take the first 80% of the team performances as the training set and validate the model on the next 20%. Using the Wilcoxon signed-rank test with confidence level 0.05, we observe that, only in 30% and 20% of ODI and Test teams, the log likelihood scores in model experiments is significantly better than the control experiments. These results suggest a significant reduction in prediction (~ 50% reduction) compared to predictability of individual performances (please see SM for more details). Further the probability of falsely accepting the null hypotheses—the control experiments perform better—increases to $\sim 10^{ - 2}$ and $\sim 10^{ - 1}$ respectively (using a binomial probability distribution with success rate 0.05 of false test result). The absence of reliable prediction in the above results suggest the absence of exploitable self-excited patterns in team performance.

### Hot winning hands

We investigate the presence of hot hands in the team performances by going through the complete history of games played by each team and analyze the winning streaks (i.e., the number of continuous wins without losing a single game in between). We note down the length of winning streaks and the corresponding frequencies of occurrences of such streaks in each team playing history.

Then, we construct a statistical ensemble of possible performance trajectories. We randomly shuffle the original performance sequences to generate *1000* synthetic performance trajectories. Using this statistical ensemble, we evaluate the null probability distribution for the joint occurrence of streaks of length *n* and of corresponding frequency *f*. We use this probability distribution for estimating the *p* values for the observed events. we define the *p* values $p( n )$ and $p( {n_{f} } )$ according to

8 $$\begin{array}{*{20}c} {p\left( n \right) = P\left( {n_{i} \ge n} \right), \quad p\left( {n_{f} } \right) = P\left( {n_{i} \ge n|f} \right)} \\ \end{array}$$

which respectively represent the *p* value for observation of streaks with length n and streaks with length n conditional on frequency *f*. To avoid the problem of multiple hypothesis testing^61^, because of simultaneous consideration of the multiple individual tests, we correct the error rates of individual tests using multiple hypothesis testing methods^62–66^. We note down the results from the methods^62–66^ and identify the extreme events (see supporting tables for multiple hypothesis testing in SM).

**Figure 5:**
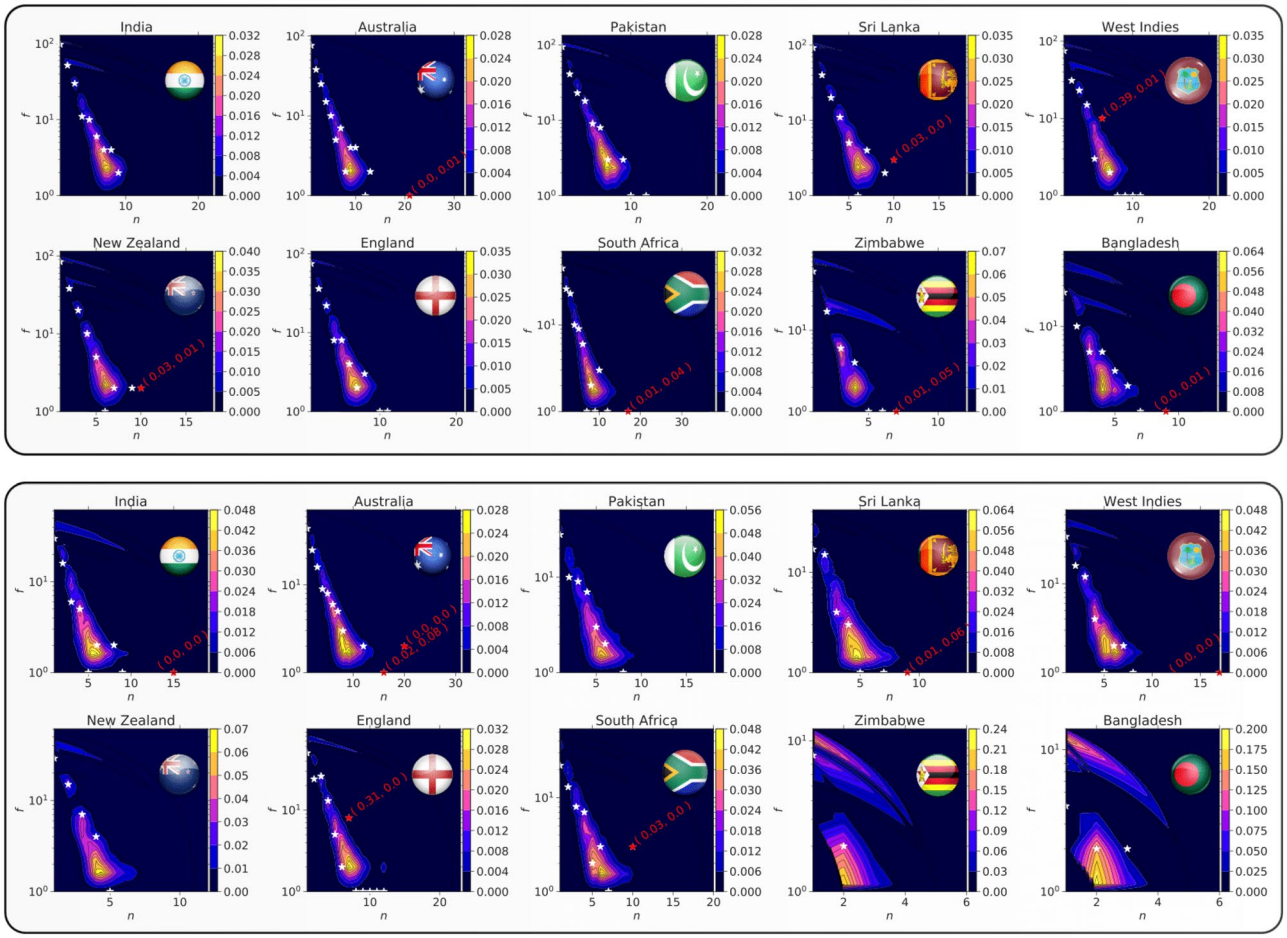
Hot hands in cricket teams. (top panel) ODI: Each of the 10 subplots in the figure shows the null distribution (obtained through randomly shuffling the performance sequence) of joint occurrence of winning streak length and of the corresponding frequency of occurrence. The title of each subplot provides the country of the team. Marked points on the plots represent the realized events. The white points represent the probable events, and the red points represent the extreme/unlikely events (determined through multiple testing methods). The *p* values ($p\left( n \right)$ and $p\left( {n_{f} } \right))$ (see Eq. (8) for definitions) for the unlikely events are provided along with the points. (Bottom panel) Test: Same as top figure for the performances in the Test format.

Figure 5 presents the position of the realized winning streaks, along with the null distribution of the winning streaks for the 10 teams in the ODI format (top panel) and in the Test format (bottom panel). The red stars in figure reveal several highly improbable i.e., one or both of $p\left( n \right)$ and $p\left( {n_{f} } \right)$ is significant with confidence level 0.05, after multiple testing. A large number of white stars indicate probable events i.e., none of $p\left( n \right)$ and $p\left( {n_{f} } \right)$ is significant. We present the $p\left( n \right)$ and $p\left( {n_{f} } \right)$ values for the events that pass the multiple hypothesis tests in figure.

We observe 5 out of 98 (5.1%) streaks in ODI cricket are significantly long, considering both their length (*n*) and frequency (f). In Test cricket, 6 out of 73 (8%) considering the length and 5 out of 73 (7%) considering the frequency are statistically significant. Because of the considered significance level, we expect an error rate of 0.05 in individual verification. In total we verified 98 possible streaks in ODI cricket and 73 streaks in Test cricket. The binomial probability for the observation of 5 hot hands in ODI cricket is 0.18 and more than 5 hot hands is 0.36. However, for the Test format, the probability of observing 5 and 6 hot hands are 0.14 and 0.08 and more than 5 and 6 are 0.15 and 0.07 respectively. This allows us to conclude that we don’t observe any Hot Hand effect in winning streaks of teams both in ODI and Test cricket. The length of winning streaks is an important variable to consider while investigating hot hands. Both the belief and the behavior of performers are sensitive to decision frames that they derive from these streak lengths^67^. This can inform coaches on the importance of how to provide information to athletes.

## Conclusion

In this study, we have quantified the predictability and persistence of individual and collective performances of the teams in a team game. We introduced a number of novel statistical tools to study the hot hand effect in a new dataset on game of Cricket. We quantified and exploited the self-excited patterns in individual and team performances to better predict the future compared to traditional methods like ARIMA.

Our investigation has confirmed the presence of significant hot-hands in individual performance. This is supported by the fact that the three highest performances in individual career cluster in time, particularly when players partake in hundreds of games. Further, the shaded branching ratios in Fig. 4A,B are very rarely found in simulated null data, confirming the strength of the self-excitation that qualifies the presence of the hot-hand effect. The major finding of our work is that these self-excitation patterns can indeed be exploited for predicting future performances. The findings of this investigation complement those of earlier studies supporting the presence of hot hands in individual careers, while raising questions about the validity of those refuting the same.

Additionally, we have showed a significant reduction in prediction of team performances compared with single players’ performance, suggesting the dominance of stochasticity in the determinant of teams’ performance. While there is still some predictability to a certain extent, the outcome of the game cannot be predicted, nor do they cluster in time. This leads us to suggest the somewhat paradoxical conclusion that ‘Cricket is a game of skill for individuals and a game of chance for the teams.’

Our study showed that, while an individual can consistently deal with the environmental systemic stochasticity, it is difficult for the team to perform equally well. Thus, these results open door for future research in the direction of the impact of group size in predictability and consistency of performance.

Furthermore, the present study established a quantitative framework for detecting and predicting the performances in individual careers. This approach will prove useful in expanding our understanding of the predictability of success in individual careers. This paper contributes to recent historiographical debates concerning the presence of hot hands in the sequence of successes in individual performances. Further work needs to be done to establish whether the presented methodology for predicting the performances can be improved for commercial usage and for financial gains, exploiting the presence of self-excited patterns in individual careers. The findings of this study have a number of important implications for future research in the field of quantifying self-excited performance patterns involved in the study of human behavior and design of algorithms for predicting success.

### Limitations

Our analysis has been performed on players with more than 30 games. Such minimum sample size is required to accurately fit the Hawkes process to data, as shown in the SM with the measures of dispersion. We acknowledge that this could have influenced the overall result as we cannot infer the existence/absence of hot hands in shorter careers. Additionally, the Hawkes point process used as a representation of the performance sequence assumes a constant background rate of new events. Thus, our methodology doesn’t account for possible seasonal variation of the performances within the players’ career. A further study could improve the methodology by considering the temporal variation of the background rate using the methods developed in^68^ to successfully account of possible complex seasonality effects. Through this study, we analyze the hot hand effect within the time frame of individual games. A natural extension of our work would be to analyze alternative time frames, such as different sections of a game (half-time, set), a half-season or season or multiple seasons to investigate the existence of hot hands.

## Supplementary Information

Supplementary Information.

## Data Availability

The datasets used in this study is publicly available at https://www.espncricinfo.com, http://howstat.com/. All methods were carried out in accordance with relevant guidelines and regulations. All data, codes, and materials used in the analysis would be made available.
