# Supplementary material for: Significant hot hand effect in the game of cricket
Source: Sci Rep. 2022 Jul 8;12:11663. doi: 10.1038/s41598-022-14980-7 (PMC9270381; doi:10.1038/s41598-022-14980-7)
Supplement: Supplementary file 1 — Supplementary Information. [file 41598_2022_14980_MOESM1_ESM.pdf]

Supplementary Materials

Data Preparation

We acquire the player and team performance dataset from `https://www.espncricinfo.com` and `http://howstat.com/` websites.

The `https://www.espncricinfo.com` website provides the complete list of cricket players, who have played at least one international cricket match, through its sub-URL `https://stats.espncricinfo.com/ci/engine/stats/index.html`. Furthermore, the website provides a graphical user interface for manually downloading and storing the list of players in HTML format. In addition to that, another sub-URL `http://search.espncricinfo.com/ci/content/player/search.html` provides a graphical user interface for accessing the information (performance in each match) about a given player and download it in HTML format. Even though the above dataset is available and accessible for public usage, doing a systematic quantitative analysis of the complete database is difficult, because of the involved accessibility problem. To solve this problem, we constructed an automated web-navigation system in python. The system sends requests to the website to obtain the dataset in HTML format with sufficient delay between consecutive requests in order to mimic a human's navigation pattern, without harming the website. We then parsed the HTML source to obtain all the useful information for our analysis and stored the structured data in a MongoDB database for the analysis. We also stored the structured data for the Team Performance, which we obtained from `http://www.howstat.com/`.

We used the web-navigation framework to browse through `http://howstat.com/` and collected the team performance dataset. We listed all the international games played between various teams, along with the dates and the outcome. Furthermore, we navigated through the scoreboards of each game to collect the total scores for each team in each game.

About the game of cricket

- In *ODI* cricket, each team gets a chance to perform (bat) once. The sequence of performance within the game is decided with a coin toss. The individuals within the teams get the chance to perform (bat) one after another to maximize the team performance. Hence the goal of each team in the game is to maximize the team performance within a limited time frame (50 overs) and limited number of dismissals (10 individuals).

- Similarly in *Test* cricket each team gets a chance to perform (bat) a maximum of two times (two innings) within a game period of maximum 5 days. The individuals within the teams get the chance to perform one after another to maximize the team performance. Hence the goal of each team in the game is to maximize the team performance within a limited number of dismissals (10 individuals) with no over restrictions.

- In both formats the team performance in can be considered as the aggregation of participating individual performances.
- **Individual performance**
  1. We look at the individual batting performances for our study.
  2. An innings is one of the divisions of a cricket match during which one team takes its turn to bat.
  3. We call the total scored runs by an individual as the performance. While doing this, we add a water level of 1 with the runs, i.e, $S_j(t) = Run_j(t) + 1$. By doing this, we set the smallest score to 1, which provides us with a well-defined performance fingerprint value for all the performance values. In other words, adding 1 removes the singularity associated with zero run scored in our performance fingerprint (1).
- **Team performance**
  1. For both *ODI* and *Test* cricket, the performance of the first batting team determines the trajectory for rest of the game. Hence, we only consider the batting performances of the first batting team for the quantification.
  2. For the analysis, we only take into account the games that had a definite outcome, i.e, Win or Loss for either of the teams. We don't study the games where there was no winning team for the match.

Supporting Results

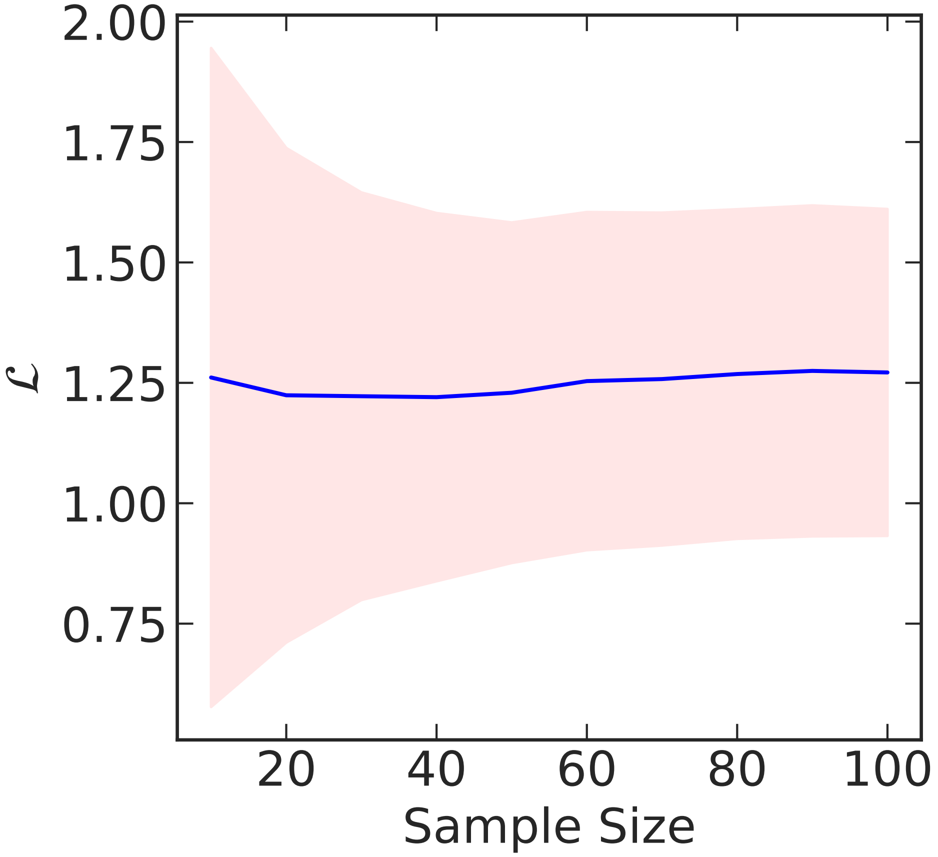

Figure S1: **Goodness of fit**: The mean (blue line) and standard deviation (red band) of log-likelihood scores is plotted against the sample size. We observe that the estimation becomes reliable close to sample size of 30.

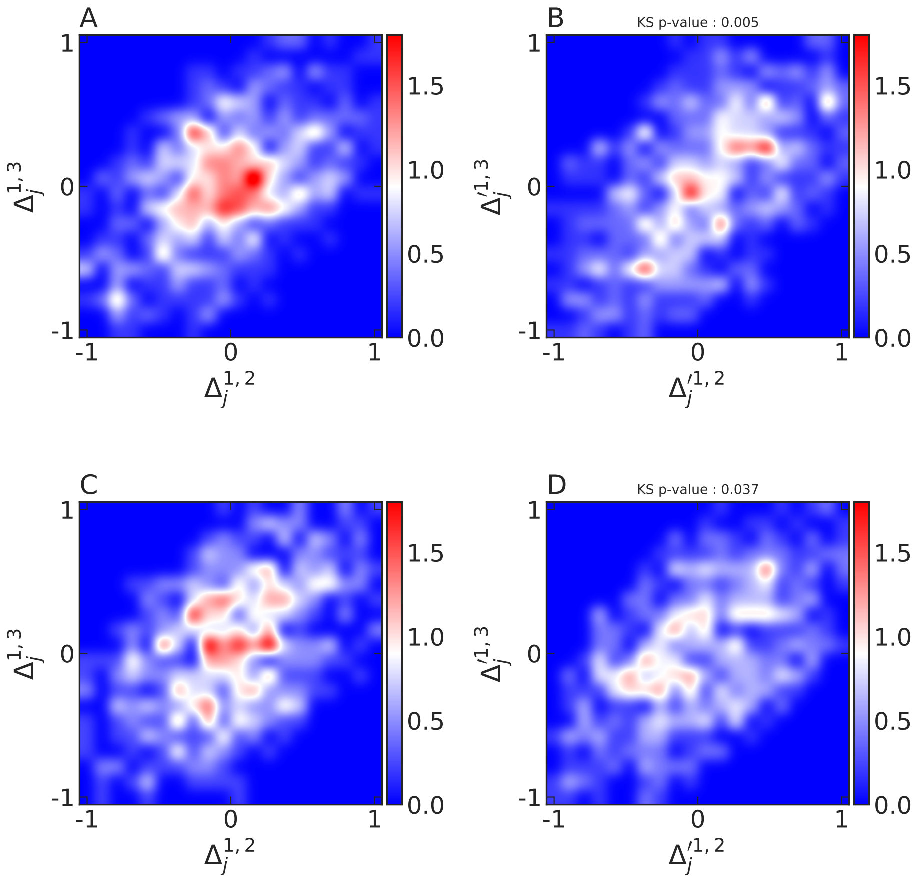

Figure S2: **Joint probability distribution $Q(\Delta_j^{1,2}, \Delta_j^{1,3})$ and the corresponding null distributions $Q(\Delta_j'^{1,2}, \Delta_j'^{1,3})$.** (A) and (B) correspond to the performances in ODI cricket and (C) and (D) correspond to the performances in Test cricket. (A) and (C) show the joint distribution of the relative difference of the indices of second best from the best, plotted against the third best from the best performances in the dataset. (B) and (D) show the joint distribution of the same quantities but the quantities are measured from randomly shuffled performance sequences in the dataset. The p-values from 2D Kolmogorov-Smirnov two sample test [2, 5] is presented in each cases.

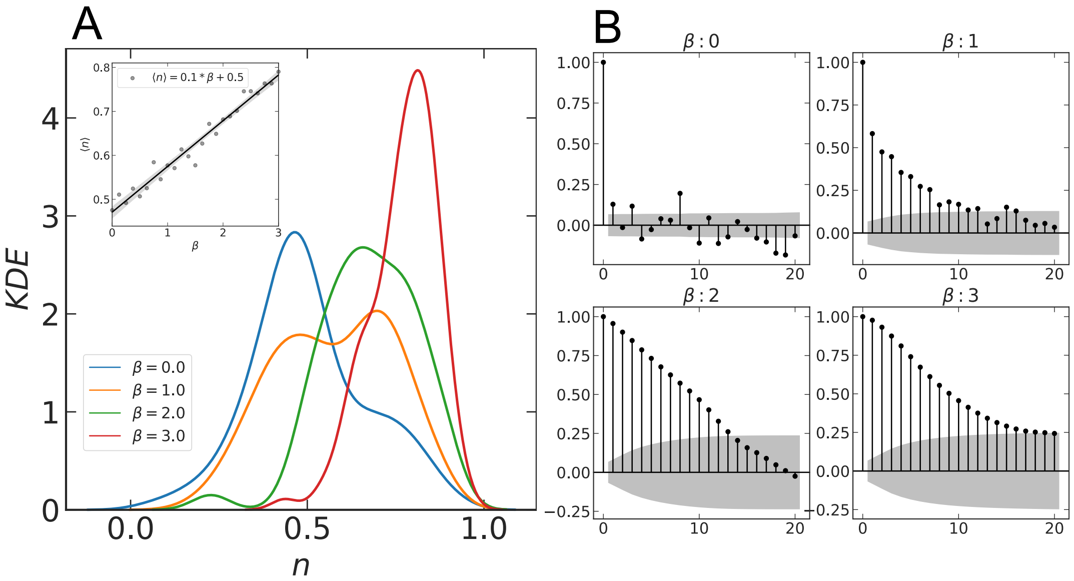

Figure S3: **Hawkes point process along the colored noise performance time**. (A) Kernel Density Estimation (KDE) of branching ratios obtained from colored noise [9]. β is the exponent of the colored noise. The inset figure represents the median value of branching ratio plotted against the exponent of the underlying colored noise. (B) The Auto Correlation Function (ACF) of the colored noise with the the given exponent values.

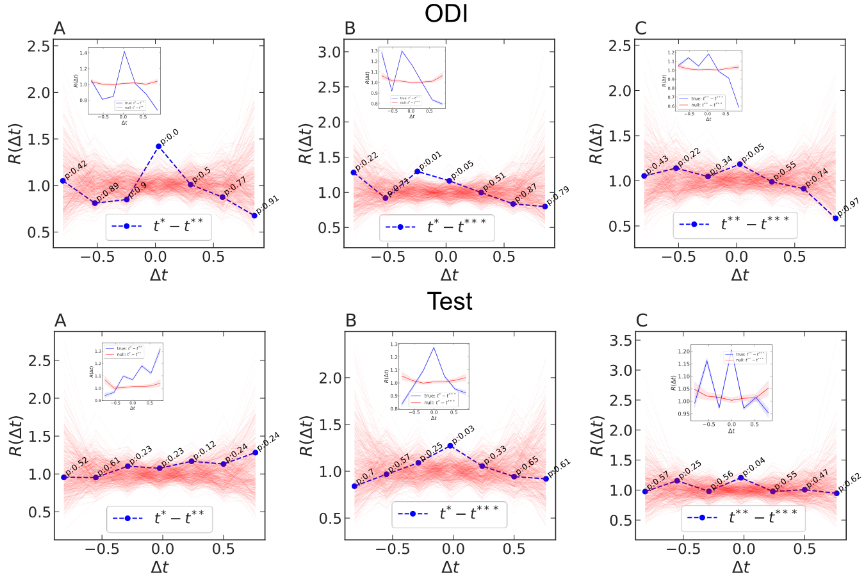

Figure S4: **Robustness of** $R(\Delta t)$: We construct 500 null $R(\Delta t)$ when the data is random and compare it with the obtained results to check the significance and robustness of the obtained results . We first reshuffle the true data to obtain a random data. We consider this random data as the pseudo true data. We reshuffle this pseudo true data to construct the corresponding null $R(\Delta t)$ (please see methods section). This gives a realisation of $R(\Delta t)$ for a time series where there is no signal. We repeat this 500 times to obtain the confidence intervals. The red lines are the 500 realizations of the null $R(\Delta t)$ signals . The blue line is the median of 500 true signals. The p-values around the center suggest the robustness of the signals. The inset figures represent the comparison of the two signals with corresponding 95% confidence intervals.

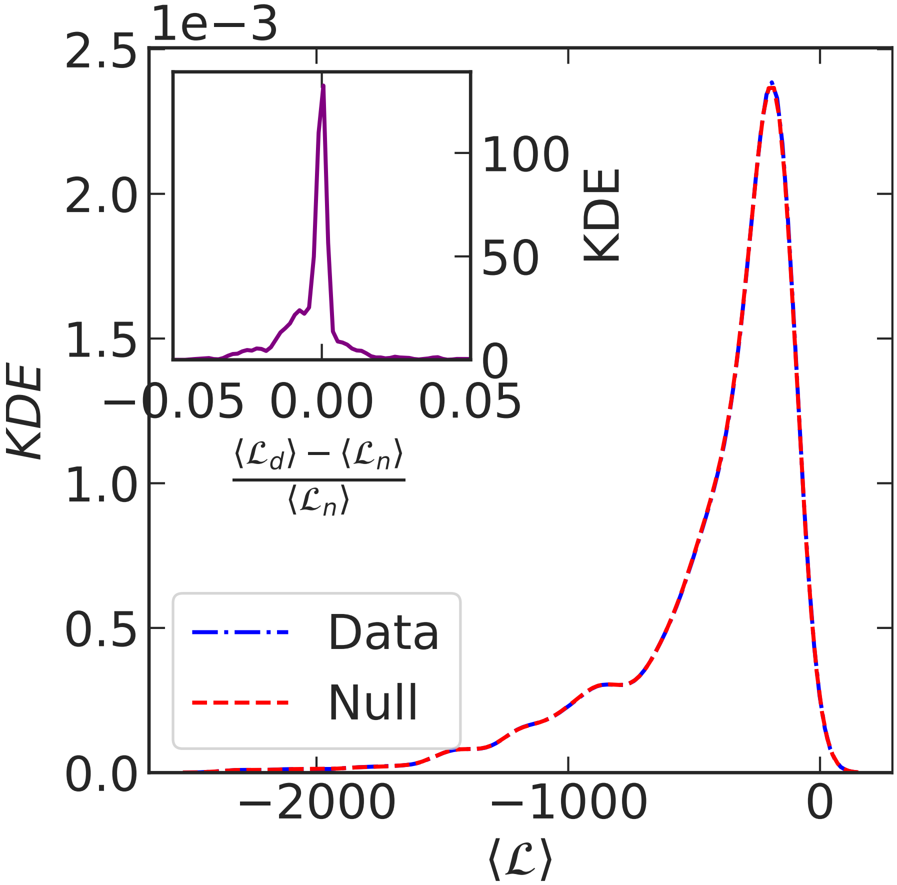

Figure S5: **ARIMA Model:** We calibrate all the individual performances in ODI cricket using the ARIMA model. We conduct differencing tests (Kwiatkowski-Phillips-Schmidt-Shin, Augmented Dickey-Fuller and Phillips-Perron) to determine the order of differencing, 'd'. We analyse the auto-correlation functions (ACF), partial auto-correlation functions (PACF) and use the Canova-Hansen method to determine the optimal value of 'p' and 'q' for each model. Where p is the number of autoregressive terms, d is the number of nonseasonal differences needed for stationarity, and q is the number of lagged forecast errors in the prediction equation. We perform the ARIMA calibration 100 times on each performance sequence and randomly shuffled performance sequence (null), and record the log-likelihood scores. The main figure represents the Kernel Density Estimation (KDE) of median log-likelihood scores obtained from the original performance sequence and the shuffled performance sequence. The inset figure shows the KDE for the relative difference of median log-likelihood scores obtained from the original performance sequence and the shuffled null performance sequence. The ARIMA model is not able to identfiy the hot hand effect, as there are no significant differences between data and the null.

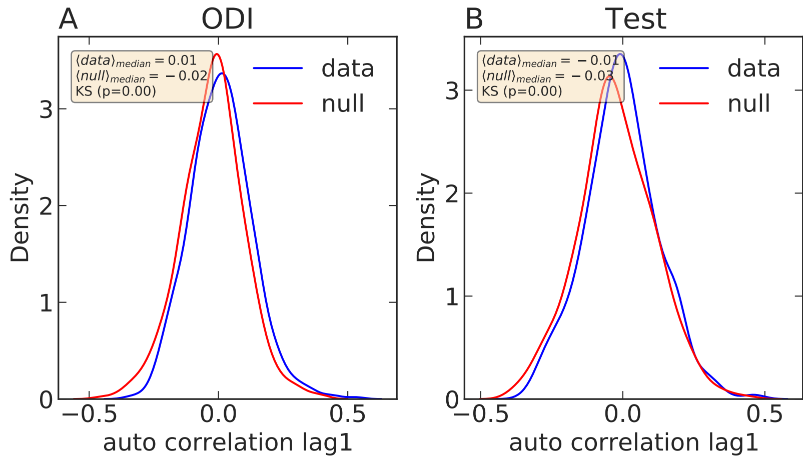

Figure S6: **Auto-correlation :** We estimate the auto-correlation values for a lag value of 1 time step, for consecutive player performances (data) and for the randomly shuffled (null) player performances. Panel A represents the kernel density of the auto-correlation values from ODI format and panel B represents the Test format. In both cases, the median value obtained from both the data and the null are around ~ 0.0. The auto-correlation metric does not allow us to identify the hot hand effect, as there are no significant differences between data and the null.

Model comparison

- We perform Shapiro-Wilk test on the the original null log-likelihood distributions for each career and find that the paired differences are not normally distributed.
- We thus perform the Wilcoxon signed-rank test to determine the significance of the observed results.
- We found that 223 out of 610 players (or 36% players) have a significantly better (with confidence level 5%) median log-likelihood score in original performance sequence compared to the shuffled sequence. Thus the probability of falsely rejecting the null hypothesis is $< 10^{-6}$. And we conclude that there is some predictable pattern hidden inside the performance sequences.
- While we compare these results with the model proposed in the main text, we find that our model produces better prediction results on 46.8% of the player compared to 36% players better prediction results with the ARIMA model. This supports the superiority of our model compared with traditional forecasting models.

Hot team hands

Team	n				$\mathcal{L}$			
	$\langle Data\rangle$	$\langle Null\rangle$	$\langle Data\rangle > \langle Null\rangle$	p	$\langle Model\rangle$	$\langle Control\rangle$	$\langle Model\rangle > \langle Control\rangle$	p
Australia	1.30E-04	5.48E-05	True	$< 10^{-6}$	4.53E+00	4.53E+00	False	$< 10^{-6}$
Bangladesh	5.58E-10	1.82E-10	True	$< 10^{-6}$	4.44E+00	4.44E+00	False	$< 10^{-6}$
England	8.55E-07	8.54E-07	True	$< 10^{-6}$	4.56E+00	4.56E+00	False	$< 10^{-6}$
India	7.63E-05	1.90E-05	True	$< 10^{-6}$	4.54E+00	4.54E+00	False	$< 10^{-6}$
New Zealand	8.73E-06	4.47E-06	True	$< 10^{-6}$	4.53E+00	4.53E+00	False	$3 \cdot 10^{-1}$
Pakistan	5.71E-05	5.13E-05	True	$6 \cdot 10^{-1}$	4.49E+00	4.49E+00	True	$< 10^{-6}$
South Africa	4.71E-08	2.56E-08	True	$< 10^{-6}$	4.58E+00	4.58E+00	False	$< 10^{-6}$
Sri Lanka	9.00E-05	1.24E-05	True	$< 10^{-6}$	4.43E+00	4.43E+00	False	$< 10^{-6}$
West Indies	6.88E-07	1.81E-06	False	$< 10^{-6}$	4.41E+00	4.41E+00	True	$< 10^{-6}$
Zimbabwe	2.56E-08	6.94E-08	False	$< 10^{-6}$	4.25E+00	4.25E+00	True	$< 10^{-6}$

Table S1: **Prediction of team performance in *ODI* format.** We partition the performances into training and validation set. We quantify the log-likelihood scores ($\mathcal{L}$) for the control and model forecasts 100 times and note down the median values. Similarly, we estimate the branching ratio (n) on the original data and shuffled data (Null), 100 times and note down the median values. We perform the Wilcoxon signed-rank test to determine the significance values for the observations. We respectively present (from left to right columns) the median value of branching ratio obtained from the data ($\langle Data\rangle$), the median value of branching ratio obtained from the randomly shuffled data ($\langle Null\rangle$), if $\langle Data\rangle > \langle Null\rangle$ and the p-value. The median value of log-likelihood score obtained from the Model ($\langle Model\rangle$), the median value of log-likelihood score obtained from the control ($\langle Control\rangle$), if $\langle Model\rangle > \langle Control\rangle$ and the p-value.

Team	n				$\mathcal{L}$			
	$\langle Data \rangle$	$\langle Null \rangle$	$\langle Data \rangle > \langle Null \rangle$	p	$\langle Model \rangle$	$\langle Control \rangle$	$\langle Model \rangle > \langle Control \rangle$	p
Australia	4.44E-05	1.22E-06	True	$< 10^{-4}$	4.72E+00	4.72E+00	False	$< 10^{-4}$
Bangladesh	1.40E-31	1.62E-31	False	$< 10^{-4}$	4.26E+00	4.26E+00	False	$5 \cdot 10^{-2}$
England	8.97E-04	6.66E-05	True	$< 10^{-4}$	4.38E+00	4.38E+00	True	$< 10^{-4}$
India	2.26E-10	9.77E-11	True	$3 \cdot 10^{-1}$	4.70E+00	4.70E+00	False	$< 10^{-4}$
NewZealand	2.30E-10	4.99E-12	True	$< 10^{-4}$	4.58E+00	4.58E+00	False	$< 10^{-4}$
Pakistan	5.06E-12	9.84E-13	True	$< 10^{-4}$	4.45E+00	4.45E+00	False	$< 10^{-4}$
SouthAfrica	1.44E-10	1.94E-13	True	$< 10^{-4}$	4.78E+00	4.78E+00	False	$< 10^{-4}$
SriLanka	1.90E-18	2.70E-19	True	$1 \cdot 10^{-1}$	4.63E+00	4.63E+00	False	$1 \cdot 10^{-1}$
WestIndies	4.51E-11	2.00E-11	True	$3 \cdot 10^{-3}$	4.51E+00	4.51E+00	True	$< 10^{-4}$
Zimbabwe	6.75E-42	7.92E-42	False	$6 \cdot 10^{-2}$	4.55E+00	4.55E+00	False	NA

Table S2: **Prediction of team performance in *Test* format.** We partition the performances into training and validation set. We quantify the log-likelihood scores ($\mathcal{L}$) for the control and model forecasts 100 times and note down the median values. Similarly, we estimate the branching ratio (n) on the original data and shuffled data (Null), 100 times and note down the median values. We perform the Wilcoxon signed-rank test to determine the significance values for the observations. We respectively present (from left to right columns) the median value of branching ratio obtained from the data ($\langle Data \rangle$), the median value of branching ratio obtained from the randomly shuffled data ($\langle Null \rangle$), if $\langle Data \rangle > \langle Null \rangle$ and the p-value. The median value of log-likelihood score obtained from the Model ($\langle Model \rangle$), the median value of log-likelihood score obtained from the control ($\langle Control \rangle$), if $\langle Model \rangle > \langle Control \rangle$ and the p-value.

Hot winning hands

Multiple Hypothesis Testing

We face the problem of multiple hypothesis testing, while simultaneously considering multiple individual tests on the same dataset or dependent datasets. In order to resolve the above problem, a number of methods have been proposed, which try to solve the issue by correcting the error rates of individual tests according to the number of simultaneously considered hypotheses as well as *p-values* for the individual tests. Below is a list of methods that we have implemented to test our hypotheses [6].

Sidak's Test

Let $p_1, \ldots, p_m$ be the *p-values* for the family of m null hypotheses $H_1, \ldots, H_m$. If we set the family-wise alpha level to α, according to the test, we reject all null hypotheses that have a *p-value* lower than $\alpha_{SID} = 1 - (1-\alpha)^{\frac{1}{m}}$. This test produces a family-wise Type I error rate of exactly α when the tests are independent from each other and all null hypotheses are true.[7]

Holm's Test

Let $p_1, \ldots, p_m$ be the *p-values* for the family of m null hypotheses $H_1, \ldots, H_m$, and let us introduce the sorted values (from lowest to highest) denoted $p_{(1)} \ldots p_{(m)}$ with the associated hypotheses be $H_{(1)}, \ldots, H_{(m)}$. Then for a given significance level α, if k is the minimal index such that $p_{(k)} > \frac{\alpha}{m+1-k}$, we reject the null hypotheses $H_{(1)}, \ldots, H_{(k-1)}$ and do not reject $H_{(k)}, \ldots, H_{(m)}$. If $k = 1$ then we do not reject any of the null hypotheses. If no such k exists, then we reject all of the null hypotheses. This method ensures that FWER $\leq \alpha$, where FWER is the family-wise error rate.[4]

Hochberg's Test

Let $p_1, \ldots, p_m$ be the *p-values* for the family of m null hypotheses $H_1, \ldots, H_m$, and let us introduce the sorted values (from lowest to highest) denoted $p_{(1)} \ldots p_{(m)}$ with the associated hypotheses be $H_{(1)}, \ldots, H_{(m)}$. For a given α , let R be the largest k such that $P_{(k)} \leq \frac{\alpha}{m-k+1}$. Then, we reject the null hypotheses $H_{(1)} \ldots H_{(R)}$.[3]

Bonferroni's Test

Let $p_1, \ldots, p_m$ be the *p-values* for the family of m null hypotheses $H_1, \ldots, H_m$, where m_0 is the number of true null hypotheses. Let the family-wise error rate *(FWER)* be the probability of rejecting at least one true H_i, that is, of making at least one type I error. Then the Bonferroni correction rejects the null hypothesis for each $p_i \leq \frac{\alpha}{m}$, thereby controlling the $FWER$ at $\leq \alpha$. [7]

Classic FDR Test

Let $p_1, \ldots, p_m$ be the *p-values* for the family of m null hypotheses $H_1, \ldots, H_m$, and let us define the sorted values (from lowest to highest) denoted $p_{(1)} \ldots p_{(m)}$ with the associated hypotheses be

$H_{(1)}, \ldots, H_{(m)}$. The Benjamini-Hochberg test controls the FDR (False Discovery Rate) at level α. For a given α, if k is the largest value such that $P_{(k)} \leq \frac{k}{m}\alpha$, we reject the null hypothesis (i.e., declare discoveries) for all $H_{(i)}$ for $i = 1, \ldots, k$. [1, 3]

Storey's Test

Let $T_1, \ldots, T_m$ be i.i.d. random variables representing the test statistics associated with m tests of the null hypothesis H_0 versus an alternative hypothesis H_i, such that

$$T_i \mid H_i \sim (1 - H_i) \cdot F_0 + H_i \cdot F_1$$

In other words, T_i follows the null distribution F_0 if $H_i = 0$, i.e, H_0 is true for test i, else T_i follows the alternative distribution F_1 if $H_i = 1$, i.e, H_1 is true. Suppose $H_i \sim \text{Bernoulli}(\pi_1)$, with probability of success for H_i and H_0 respectively π_1 and $\pi_0 = 1 - \pi_1$. We denote the critical region at significance level α by Γ_α, corresponding to the values of T_i for which H_0 is rejected beyond Γ_α. Let an experiment yield a value t for the test statistic. The q-value of t is formally defined as

$$\inf_{\{\Gamma_\alpha : t \in \Gamma_\alpha\}} \text{pFDR}(\Gamma_\alpha)$$

That is, the *q-value* is the infimum of the pFDR if H_0 is rejected for test statistics with values $\geq t$. Equivalently, the q-value equals

$$\inf_{\{\Gamma_\alpha : t \in \Gamma_\alpha\}} \Pr(H = 0 \mid T \in \Gamma_\alpha)$$

which is the infimum of the probability that H_0 is true given that H_0 is rejected (the false discovery rate). We use the *q-value* to reject or accept the null hypothesis.[8]

Winning Streaks in ODI Teams

Country	n	f	$p(n)$	$p(n_f)$	Sidak		Holm		Hochberg		Bonferroni		Classic FDR		Storey FDR		Storey's Q	
.	.	.	.	.	$p(n)$	$p(n_f)$	$p(n)$	$p(n_f)$	$p(n)$	$p(n_f)$	$p(n)$	$p(n_f)$	$p(n)$	$p(n_f)$	$p(n)$	$p(n_f)$	$p(n)$	$p(n_f)$
AUS	1	76	1.00	1.00	False	False	False	False	False	False	False	False	False	False	False	False	1.00	1.00
AUS	2	38	0.91	1.00	False	False	False	False	False	False	False	False	False	False	False	False	1.00	1.00
AUS	3	25	0.82	1.00	False	False	False	False	False	False	False	False	False	False	False	False	1.00	1.00
AUS	4	15	0.73	0.98	False	False	False	False	False	False	False	False	False	False	False	False	0.98	0.98
AUS	5	10	0.65	0.96	False	False	False	False	False	False	False	False	False	False	False	False	0.96	0.96
AUS	6	5	0.56	0.97	False	False	False	False	False	False	False	False	False	False	False	False	0.97	0.97
AUS	7	7	0.47	0.40	False	False	False	False	False	False	False	False	False	False	False	False	0.31	0.31
AUS	8	2	0.38	0.92	False	False	False	False	False	False	False	False	False	False	False	False	0.76	0.92
AUS	9	4	0.30	0.20	False	False	False	False	False	False	False	False	False	False	False	False	0.07	0.07
AUS	10	4	0.22	0.07	False	False	False	False	False	False	False	False	False	False	False	False	0.22	0.15
AUS	12	1	0.11	0.47	False	False	False	False	False	False	False	False	False	False	**True**	False	0.03	0.06
AUS	13	2	0.07	0.05	False	False	False	False	False	False	False	False	False	False	False	False	0.07	0.07
AUS	21	1	0.00	0.01	**True**	**True**	**True**	**True**	**True**	**True**	**True**	**True**	**True**	**True**	**True**	**True**	0.00	0.01

Table S3: Winning streak statistics for team Australia in *ODI* format. In the table n denotes the length of the winning streak, f is the corresponding frequency of occurrence. $p(n)$ and $p(n_f)$ are the *p-values* for observation of streaks with length n and streaks with length n conditional on frequency f. With the help of multiple hypothesis tests mentioned in the previous section, we check the significance of the *p-values* and note down the results. We write result to be True, for a positive result, associated with a significant *p-value* below the standard confidence level of 0.05, otherwise it is False.

Country	n	f	$p(n)$	$p(n_f)$	Sidak		Holm		Hochberg		Bonferroni		Classic FDR		Storey FDR		Storey's Q	
.	.	.	.	.	$p(n)$	$p(n_f)$	$p(n)$	$p(n_f)$	$p(n)$	$p(n_f)$	$p(n)$	$p(n_f)$	$p(n)$	$p(n_f)$	$p(n)$	$p(n_f)$	$p(n)$	$p(n_f)$
BAN	1	25	1.00	1.00	False	False	False	False	False	False	False	False	False	False	False	False	1.00	1.00
BAN	2	10	0.79	1.00	False	False	False	False	False	False	False	False	False	False	False	False	1.00	1.00
BAN	3	5	0.58	1.00	False	False	False	False	False	False	False	False	False	False	False	False	1.00	1.00
BAN	4	5	0.37	0.18	False	False	False	False	False	False	False	False	False	False	False	False	0.11	0.11
BAN	5	3	0.18	0.12	False	False	False	False	False	False	False	False	False	False	False	False	0.18	0.18
BAN	6	2	0.07	0.05	False	False	False	False	False	False	False	False	False	False	False	False	0.07	0.07
BAN	7	1	0.02	0.12	**True**	False	False	False	**True**	False	**True**	False	**True**	False	**True**	False	0.05	0.12
BAN	9	1	0.00	0.01	**True**	**True**	**True**	**True**	**True**	**True**	**True**	**True**	**True**	**True**	**True**	**True**	0.01	0.01

Table S4: Winning streak statistics for team Bangladesh in *ODI* format. In the table n denotes the length of the winning streak, f is the corresponding frequency of occurrence. $p(n)$ and $p(n_f)$ are the *p-values* for observation of streaks with length n and streaks with length n conditional on frequency f. With the help of multiple hypothesis tests mentioned in the previous section, we check the significance of the *p-values* and note down the results. We write result to be True, for a positive result, associated with a significant *p-value* below the standard confidence level of 0.05, otherwise it is False.

Country	n	f	$p(n)$	$p(n_f)$	Sidak		Holm		Hochberg		Bonferroni		Classic FDR		Storey FDR		Storey's Q	
.	.	.	.	.	$p(n)$	$p(n_f)$	$p(n)$	$p(n_f)$	$p(n)$	$p(n_f)$	$p(n)$	$p(n_f)$	$p(n)$	$p(n_f)$	$p(n)$	$p(n_f)$	$p(n)$	$p(n_f)$
ENG	1	75	1.00	1.00	False	False	False	False	False	False	False	False	False	False	False	False	1.00	1.00
ENG	2	36	0.88	1.00	False	False	False	False	False	False	False	False	False	False	False	False	1.00	1.00
ENG	3	22	0.76	1.00	False	False	False	False	False	False	False	False	False	False	False	False	1.00	1.00
ENG	4	8	0.64	1.00	False	False	False	False	False	False	False	False	False	False	False	False	1.00	1.00
ENG	5	8	0.52	0.68	False	False	False	False	False	False	False	False	False	False	False	False	0.68	0.68
ENG	6	4	0.40	0.70	False	False	False	False	False	False	False	False	False	False	False	False	0.70	0.70
ENG	7	2	0.28	0.71	False	False	False	False	False	False	False	False	False	False	False	False	0.57	0.71
ENG	8	3	0.19	0.12	False	False	False	False	False	False	False	False	False	False	False	False	0.19	0.19
ENG	10	1	0.06	0.29	False	False	False	False	False	False	False	False	False	False	**True**	**True**	0.01	0.02
ENG	11	1	0.03	0.17	False	False	False	False	False	False	False	False	False	False	False	False	0.07	0.17

Table S5: Winning streak statistics for team England in *ODI* format. In the table n denotes the length of the winning streak, f is the corresponding frequency of occurrence. $p(n)$ and $p(n_f)$ are the *p-values* for observation of streaks with length n and streaks with length n conditional on frequency f. With the help of multiple hypothesis tests mentioned in the previous section, we check the significance of the *p-values* and note down the results. We write result to be True, for a positive result, associated with a significant *p-value* below the standard confidence level of 0.05, otherwise it is False.

Country	n	f	$p(n)$	$p(n_f)$	Sidak		Holm		Hochberg		Bonferroni		Classic FDR		Storey FDR		Storey's Q	
.	.	.	.	.	$p(n)$	$p(n_f)$	$p(n)$	$p(n_f)$	$p(n)$	$p(n_f)$	$p(n)$	$p(n_f)$	$p(n)$	$p(n_f)$	$p(n)$	$p(n_f)$	$p(n)$	$p(n_f)$
IND	1	98	1.00	1.00	False	False	False	False	False	False	False	False	False	False	False	False	1.00	1.00
IND	2	52	0.89	1.00	False	False	False	False	False	False	False	False	False	False	False	False	1.00	1.00
IND	3	30	0.78	1.00	False	False	False	False	False	False	False	False	False	False	False	False	1.00	1.00
IND	4	11	0.67	1.00	False	False	False	False	False	False	False	False	False	False	False	False	1.00	1.00
IND	5	10	0.56	0.90	False	False	False	False	False	False	False	False	False	False	False	False	0.90	0.90
IND	6	6	0.45	0.70	False	False	False	False	False	False	False	False	False	False	False	False	0.70	0.70
IND	7	4	0.34	0.47	False	False	False	False	False	False	False	False	False	False	False	False	0.28	0.28
IND	8	4	0.24	0.10	False	False	False	False	False	False	False	False	False	False	False	False	0.24	0.20
IND	9	2	0.15	0.30	False	False	False	False	False	False	False	False	False	False	**True**	**True**	0.04	0.04

Table S6: Winning streak statistics for team India in *ODI* format. In the table n denotes the length of the winning streak, f is the corresponding frequency of occurrence. $p(n)$ and $p(n_f)$ are the *p-values* for observation of streaks with length n and streaks with length n conditional on frequency f. With the help of multiple hypothesis tests mentioned in the previous section, we check the significance of the *p-values* and note down the results. We write result to be True, for a positive result, associated with a significant *p-value* below the standard confidence level of 0.05, otherwise it is False.

Country	n	f	$p(n)$	$p(n_f)$	Sidak		Holm		Hochberg		Bonferroni		Classic FDR		Storey FDR		Storey's Q	
.	.	.	.	.	$p(n)$	$p(n_f)$	$p(n)$	$p(n_f)$	$p(n)$	$p(n_f)$	$p(n)$	$p(n_f)$	$p(n)$	$p(n_f)$	$p(n)$	$p(n_f)$	$p(n)$	$p(n_f)$
NZL	1	84	1.00	1.00	False	False	False	False	False	False	False	False	False	False	False	False	1.00	1.00
NZL	2	38	0.87	1.00	False	False	False	False	False	False	False	False	False	False	False	False	1.00	1.00
NZL	3	20	0.73	1.00	False	False	False	False	False	False	False	False	False	False	False	False	1.00	1.00
NZL	4	10	0.60	0.99	False	False	False	False	False	False	False	False	False	False	False	False	0.99	0.99
NZL	5	5	0.46	0.91	False	False	False	False	False	False	False	False	False	False	False	False	0.91	0.91
NZL	6	1	0.33	0.98	False	False	False	False	False	False	False	False	False	False	False	False	0.66	0.98
NZL	7	2	0.21	0.44	False	False	False	False	False	False	False	False	False	False	False	False	0.15	0.16
NZL	9	2	0.06	0.03	False	False	False	False	False	False	False	False	False	False	False	False	0.06	0.06
NZL	10	2	0.03	0.01	False	**True**	False	**True**	**True**	**True**	False	**True**	**True**	**True**	**True**	**True**	0.03	0.02

Table S7: Winning streak statistics for team New Zealand in *ODI* format. In the table n denotes the length of the winning streak, f is the corresponding frequency of occurrence. $p(n)$ and $p(n_f)$ are the *p-values* for observation of streaks with length n and streaks with length n conditional on frequency f. With the help of multiple hypothesis tests mentioned in the previous section, we check the significance of the *p-values* and note down the results. We write result to be True, for a positive result, associated with a significant *p-value* below the standard confidence level of 0.05, otherwise it is False.

Country	n	f	$p(n)$	$p(n_f)$	Sidak		Holm		Hochberg		Bonferroni		Classic FDR		Storey FDR		Storey's Q	
.	.	.	.	.	$p(n)$	$p(n_f)$	$p(n)$	$p(n_f)$	$p(n)$	$p(n_f)$	$p(n)$	$p(n_f)$	$p(n)$	$p(n_f)$	$p(n)$	$p(n_f)$	$p(n)$	$p(n_f)$
PAK	1	94	1.00	1.00	False	False	False	False	False	False	False	False	False	False	False	False	1.00	1.00
PAK	2	41	0.89	1.00	False	False	False	False	False	False	False	False	False	False	False	False	1.00	1.00
PAK	3	23	0.78	1.00	False	False	False	False	False	False	False	False	False	False	False	False	1.00	1.00
PAK	4	18	0.66	0.95	False	False	False	False	False	False	False	False	False	False	False	False	0.95	0.95
PAK	5	9	0.55	0.89	False	False	False	False	False	False	False	False	False	False	False	False	0.89	0.89
PAK	6	8	0.44	0.22	False	False	False	False	False	False	False	False	False	False	False	False	0.17	0.17
PAK	7	3	0.33	0.65	False	False	False	False	False	False	False	False	False	False	False	False	0.65	0.65
PAK	9	3	0.14	0.05	False	False	False	False	False	False	False	False	False	False	False	False	0.14	0.10
PAK	10	1	0.09	0.41	False	False	False	False	False	False	False	False	False	False	**True**	False	0.04	0.08
PAK	12	1	0.03	0.14	False	False	False	False	False	False	False	False	False	False	False	False	0.05	0.14

Table S8: Winning streak statistics for team Pakistan in *ODI* format. In the table n denotes the length of the winning streak, f is the corresponding frequency of occurrence. $p(n)$ and $p(n_f)$ are the *p-values* for observation of streaks with length n and streaks with length n conditional on frequency f. With the help of multiple hypothesis tests mentioned in the previous section, we check the significance of the *p-values* and note down the results. We write result to be True, for a positive result, associated with a significant *p-value* below the standard confidence level of 0.05, otherwise it is False.

Country	n	f	$p(n)$	$p(n_f)$	Sidak		Holm		Hochberg		Bonferroni		Classic FDR		Storey FDR		Storey's Q	
.	.	.	.	.	$p(n)$	$p(n_f)$	$p(n)$	$p(n_f)$	$p(n)$	$p(n_f)$	$p(n)$	$p(n_f)$	$p(n)$	$p(n_f)$	$p(n)$	$p(n_f)$	$p(n)$	$p(n_f)$
SAF	1	45	1.00	1.00	False	False	False	False	False	False	False	False	False	False	False	False	1.00	1.00
SAF	2	26	0.90	1.00	False	False	False	False	False	False	False	False	False	False	False	False	1.00	1.00
SAF	3	23	0.81	0.79	False	False	False	False	False	False	False	False	False	False	False	False	0.81	0.81
SAF	4	10	0.71	0.98	False	False	False	False	False	False	False	False	False	False	False	False	0.98	0.98
SAF	5	9	0.62	0.71	False	False	False	False	False	False	False	False	False	False	False	False	0.71	0.71
SAF	6	6	0.52	0.69	False	False	False	False	False	False	False	False	False	False	False	False	0.69	0.69
SAF	7	1	0.43	0.99	False	False	False	False	False	False	False	False	False	False	False	False	0.86	0.99
SAF	8	2	0.34	0.76	False	False	False	False	False	False	False	False	False	False	False	False	0.67	0.76
SAF	9	1	0.25	0.83	False	False	False	False	False	False	False	False	False	False	False	False	0.50	0.83
SAF	10	3	0.18	0.09	False	False	False	False	False	False	False	False	False	False	False	False	0.18	0.18
SAF	12	1	0.08	0.34	False	False	False	False	False	False	False	False	False	False	**True**	False	0.02	0.05
SAF	17	1	0.01	0.04	**True**	False	**True**	False	**True**	**True**	**True**	False	**True**	**True**	**True**	**True**	0.02	0.04

Table S9: Winning streak statistics for team South Africa in *ODI* format. In the table n denotes the length of the winning streak, f is the corresponding frequency of occurrence. $p(n)$ and $p(n_f)$ are the *p-values* for observation of streaks with length n and streaks with length n conditional on frequency f. With the help of multiple hypothesis tests mentioned in the previous section, we check the significance of the *p-values* and note down the results. We write result to be True, for a positive result, associated with a significant *p-value* below the standard confidence level of 0.05, otherwise it is False.

Country	n	f	$p(n)$	$p(n_f)$	Sidak		Holm		Hochberg		Bonferroni		Classic FDR		Storey FDR		Storey's Q	
.	.	.	.	.	$p(n)$	$p(n_f)$	$p(n)$	$p(n_f)$	$p(n)$	$p(n_f)$	$p(n)$	$p(n_f)$	$p(n)$	$p(n_f)$	$p(n)$	$p(n_f)$	$p(n)$	$p(n_f)$
SRL	1	92	1.00	1.00	False	False	False	False	False	False	False	False	False	False	False	False	1.00	1.00
SRL	2	40	0.87	1.00	False	False	False	False	False	False	False	False	False	False	False	False	1.00	1.00
SRL	3	20	0.74	1.00	False	False	False	False	False	False	False	False	False	False	False	False	1.00	1.00
SRL	4	11	0.60	0.97	False	False	False	False	False	False	False	False	False	False	False	False	0.97	0.97
SRL	5	5	0.47	0.94	False	False	False	False	False	False	False	False	False	False	False	False	0.94	0.94
SRL	6	1	0.34	0.99	False	False	False	False	False	False	False	False	False	False	False	False	0.68	0.99
SRL	7	4	0.22	0.10	False	False	False	False	False	False	False	False	False	False	False	False	0.22	0.21
SRL	9	2	0.06	0.05	False	False	False	False	False	False	False	False	False	False	False	False	0.06	0.06
SRL	10	3	0.03	0.00	False	**True**	False	**True**	**True**	**True**	False	**True**	**True**	**True**	**True**	**True**	0.03	0.00

Table S10: Winning streak statistics for team Sri Lanka in *ODI* format. In the table n denotes the length of the winning streak, f is the corresponding frequency of occurrence. $p(n)$ and $p(n_f)$ are the *p-values* for observation of streaks with length n and streaks with length n conditional on frequency f. With the help of multiple hypothesis tests mentioned in the previous section, we check the significance of the *p-values* and note down the results. We write result to be True, for a positive result, associated with a significant *p-value* below the standard confidence level of 0.05, otherwise it is False.

Country	n	f	$p(n)$	$p(n_f)$	Sidak		Holm		Hochberg		Bonferroni		Classic FDR		Storey FDR		Storey's Q	
.	.	.	.	.	$p(n)$	$p(n_f)$	$p(n)$	$p(n_f)$	$p(n)$	$p(n_f)$	$p(n)$	$p(n_f)$	$p(n)$	$p(n_f)$	$p(n)$	$p(n_f)$	$p(n)$	$p(n_f)$
WIN	1	75	1.00	1.00	False	False	False	False	False	False	False	False	False	False	False	False	1.00	1.00
WIN	2	31	0.88	1.00	False	False	False	False	False	False	False	False	False	False	False	False	1.00	1.00
WIN	3	23	0.76	1.00	False	False	False	False	False	False	False	False	False	False	False	False	1.00	1.00
WIN	4	15	0.63	0.90	False	False	False	False	False	False	False	False	False	False	False	False	0.63	0.63
WIN	5	3	0.51	1.00	False	False	False	False	False	False	False	False	False	False	False	False	1.00	1.00
WIN	6	10	0.39	0.01	False	**True**	False	**True**	False	**True**	False	**True**	False	**True**	False	**True**	0.12	0.00
WIN	7	2	0.27	0.72	False	False	False	False	False	False	False	False	False	False	False	False	0.54	0.72
WIN	8	1	0.17	0.70	False	False	False	False	False	False	False	False	False	False	False	False	0.34	0.70
WIN	9	1	0.10	0.45	False	False	False	False	False	False	False	False	False	False	**True**	False	0.03	0.08
WIN	10	1	0.06	0.28	False	False	False	False	False	False	False	False	False	False	**True**	**True**	0.01	0.01
WIN	11	1	0.03	0.16	False	False	False	False	False	False	False	False	False	False	False	False	0.06	0.16

Table S11: Winning streak statistics for team West Indies in *ODI* format. In the table n denotes the length of the winning streak, f is the corresponding frequency of occurrence. $p(n)$ and $p(n_f)$ are the *p-values* for observation of streaks with length n and streaks with length n conditional on frequency f. With the help of multiple hypothesis tests mentioned in the previous section, we check the significance of the *p-values* and note down the results. We write result to be True, for a positive result, associated with a significant *p-value* below the standard confidence level of 0.05, otherwise it is False.

Country	n	f	$p(n)$	$p(n_f)$	Sidak		Holm		Hochberg		Bonferroni		Classic FDR		Storey FDR		Storey's Q	
.	.	.	.	.	$p(n)$	$p(n_f)$	$p(n)$	$p(n_f)$	$p(n)$	$p(n_f)$	$p(n)$	$p(n_f)$	$p(n)$	$p(n_f)$	$p(n)$	$p(n_f)$	$p(n)$	$p(n_f)$
ZIM	1	53	1.00	1.00	False	False	False	False	False	False	False	False	False	False	False	False	1.00	1.00
ZIM	2	17	0.77	1.00	False	False	False	False	False	False	False	False	False	False	False	False	1.00	1.00
ZIM	3	6	0.53	1.00	False	False	False	False	False	False	False	False	False	False	False	False	1.00	1.00
ZIM	4	4	0.30	0.23	False	False	False	False	False	False	False	False	False	False	False	False	0.10	0.10
ZIM	5	1	0.12	0.55	False	False	False	False	False	False	False	False	False	False	False	False	0.23	0.55
ZIM	6	1	0.03	0.19	False	False	False	False	False	False	False	False	False	False	False	False	0.07	0.19
ZIM	7	1	0.01	0.05	**True**	False	**True**	False	**True**	False	**True**	False	**True**	False	**True**	False	0.02	0.05

Table S12: Winning streak statistics for team Zimbabwe in *ODI* format. In the table n denotes the length of the winning streak, f is the corresponding frequency of occurrence. $p(n)$ and $p(n_f)$ are the *p-values* for observation of streaks with length n and streaks with length n conditional on frequency f. With the help of multiple hypothesis tests mentioned in the previous section, we check the significance of the *p-values* and note down the results. We write result to be True, for a positive result, associated with a significant *p-value* below the standard confidence level of 0.05, otherwise it is False.

Winning Streaks in Test Teams

Country	n	f	$p(n)$	$p(n_f)$	Sidak		Holm		Hochberg		Bonferroni		Classic FDR		Storey FDR		Storey's Q	
.	.	.	.	.	$p(n)$	$p(n_f)$	$p(n)$	$p(n_f)$	$p(n)$	$p(n_f)$	$p(n)$	$p(n_f)$	$p(n)$	$p(n_f)$	$p(n)$	$p(n_f)$	$p(n)$	$p(n_f)$
AUS	1	44	1.00	1.00	False	False	False	False	False	False	False	False	False	False	False	False	1.00	1.00
AUS	2	25	0.91	1.00	False	False	False	False	False	False	False	False	False	False	False	False	1.00	1.00
AUS	3	16	0.81	0.99	False	False	False	False	False	False	False	False	False	False	False	False	0.99	0.99
AUS	4	9	0.72	1.00	False	False	False	False	False	False	False	False	False	False	False	False	1.00	1.00
AUS	5	8	0.62	0.85	False	False	False	False	False	False	False	False	False	False	False	False	0.85	0.85
AUS	6	6	0.53	0.72	False	False	False	False	False	False	False	False	False	False	False	False	0.72	0.72
AUS	7	5	0.43	0.40	False	False	False	False	False	False	False	False	False	False	False	False	0.30	0.30
AUS	8	3	0.34	0.52	False	False	False	False	False	False	False	False	False	False	False	False	0.23	0.23
AUS	12	2	0.08	0.07	False	False	False	False	False	False	False	False	False	False	False	False	0.08	0.08
AUS	16	1	0.02	0.08	**True**	False	**True**	False	**True**	False	**True**	False	**True**	False	**True**	False	0.03	0.08
AUS	20	2	0.00	0.00	**True**	**True**	**True**	**True**	**True**	**True**	**True**	**True**	**True**	**True**	**True**	**True**	0.00	0.00

Table S13: Winning streak statistics for team Australia in *Test* format. In the table n denotes the length of the winning streak, f is the corresponding frequency of occurrence. $p(n)$ and $p(n_f)$ are the *p-values* for observation of streaks with length n and streaks with length n conditional on frequency f. With the help of multiple hypothesis tests mentioned in the previous section, we check the significance of the *p-values* and note down the results. We write result to be True, for a positive result, associated with a significant *p-value* below the standard confidence level of 0.05, otherwise it is False.

Country	n	f	$p(n)$	$p(n_f)$	Sidak		Holm		Hochberg		Bonferroni		Classic FDR		Storey FDR		Storey's Q	
.	.	.	.	.	$p(n)$	$p(n_f)$	$p(n)$	$p(n_f)$	$p(n)$	$p(n_f)$	$p(n)$	$p(n_f)$	$p(n)$	$p(n_f)$	$p(n)$	$p(n_f)$	$p(n)$	$p(n_f)$
BAN	1	4	1.0	1.00	False	False	False	False	False	False	False	False	False	False	False	False	1.0	1.00
BAN	2	2	0.5	1.00	False	False	False	False	False	False	False	False	False	False	False	False	1.0	1.00
BAN	3	2	0.1	0.04	False	False	False	False	False	False	False	False	False	False	False	False	0.1	0.08

Table S14: Winning streak statistics for team Bangladesh in *Test* format. In the table n denotes the length of the winning streak, f is the corresponding frequency of occurrence. $p(n)$ and $p(n_f)$ are the *p-values* for observation of streaks with length n and streaks with length n conditional on frequency f. With the help of multiple hypothesis tests mentioned in the previous section, we check the significance of the *p-values* and note down the results. We write result to be True, for a positive result, associated with a significant *p-value* below the standard confidence level of 0.05, otherwise it is False.

Country	n	f	$p(n)$	$p(n_f)$	Sidak		Holm		Hochberg		Bonferroni		Classic FDR		Storey FDR		Storey's Q	
.	.	.	.	.	$p(n)$	$p(n_f)$	$p(n)$	$p(n_f)$	$p(n)$	$p(n_f)$	$p(n)$	$p(n_f)$	$p(n)$	$p(n_f)$	$p(n)$	$p(n_f)$	$p(n)$	$p(n_f)$
ENG	1	50	1.00	1.00	False	False	False	False	False	False	False	False	False	False	False	False	1.00	1.00
ENG	2	24	0.89	1.00	False	False	False	False	False	False	False	False	False	False	False	False	1.00	1.00
ENG	3	26	0.77	0.99	False	False	False	False	False	False	False	False	False	False	False	False	0.99	0.99
ENG	4	13	0.66	0.97	False	False	False	False	False	False	False	False	False	False	False	False	0.97	0.97
ENG	5	5	0.54	0.98	False	False	False	False	False	False	False	False	False	False	False	False	0.98	0.98
ENG	6	2	0.43	0.97	False	False	False	False	False	False	False	False	False	False	False	False	0.85	0.97
ENG	7	8	0.31	0.00	False	**True**	False	**True**	False	**True**	False	**True**	False	**True**	False	**True**	0.09	0.00
ENG	8	1	0.21	0.78	False	False	False	False	False	False	False	False	False	False	False	False	0.42	0.78
ENG	9	1	0.13	0.56	False	False	False	False	False	False	False	False	False	False	False	False	0.26	0.56
ENG	10	1	0.08	0.38	False	False	False	False	False	False	False	False	False	False	**True**	False	0.03	0.07
ENG	11	1	0.05	0.22	False	False	False	False	False	False	False	False	False	False	False	False	0.09	0.22
ENG	12	1	0.02	0.12	**True**	False	False	False	**True**	False	**True**	False	**True**	False	**True**	False	0.05	0.12

Table S15: Winning streak statistics for team England in *Test* format. In the table n denotes the length of the winning streak, f is the corresponding frequency of occurrence. $p(n)$ and $p(n_f)$ are the *p-values* for observation of streaks with length n and streaks with length n conditional on frequency f. With the help of multiple hypothesis tests mentioned in the previous section, we check the significance of the *p-values* and note down the results. We write result to be True, for a positive result, associated with a significant *p-value* below the standard confidence level of 0.05, otherwise it is False.

Country	n	f	$p(n)$	$p(n_f)$	Sidak		Holm		Hochberg		Bonferroni		Classic FDR		Storey FDR		Storey's Q	
.	.	.	.	.	$p(n)$	$p(n_f)$	$p(n)$	$p(n_f)$	$p(n)$	$p(n_f)$	$p(n)$	$p(n_f)$	$p(n)$	$p(n_f)$	$p(n)$	$p(n_f)$	$p(n)$	$p(n_f)$
IND	1	30	1.00	1.00	False	False	False	False	False	False	False	False	False	False	False	False	1.00	1.00
IND	2	16	0.84	1.00	False	False	False	False	False	False	False	False	False	False	False	False	1.00	1.00
IND	3	6	0.69	1.00	False	False	False	False	False	False	False	False	False	False	False	False	1.00	1.00
IND	4	5	0.53	0.87	False	False	False	False	False	False	False	False	False	False	False	False	0.87	0.87
IND	5	1	0.38	0.98	False	False	False	False	False	False	False	False	False	False	False	False	0.76	0.98
IND	6	2	0.24	0.48	False	False	False	False	False	False	False	False	False	False	False	False	0.17	0.17
IND	8	2	0.07	0.04	False	False	False	False	False	False	False	False	False	False	False	False	0.07	0.07
IND	9	1	0.03	0.15	False	False	False	False	False	False	False	False	False	False	False	False	0.07	0.15
IND	15	1	0.00	0.00	**True**	**True**	**True**	**True**	**True**	**True**	**True**	**True**	**True**	**True**	**True**	**True**	0.00	0.00

Table S16: Winning streak statistics for team India in *Test* format. In the table n denotes the length of the winning streak, f is the corresponding frequency of occurrence. $p(n)$ and $p(n_f)$ are the *p-values* for observation of streaks with length n and streaks with length n conditional on frequency f. With the help of multiple hypothesis tests mentioned in the previous section, we check the significance of the *p-values* and note down the results. We write result to be True, for a positive result, associated with a significant *p-value* below the standard confidence level of 0.05, otherwise it is False.

Country	n	f	$p(n)$	$p(n_f)$	Sidak		Holm		Hochberg		Bonferroni		Classic FDR		Storey FDR		Storey's Q	
.	.	.	.	.	$p(n)$	$p(n_f)$	$p(n)$	$p(n_f)$	$p(n)$	$p(n_f)$	$p(n)$	$p(n_f)$	$p(n)$	$p(n_f)$	$p(n)$	$p(n_f)$	$p(n)$	$p(n_f)$
NZL	1	29	1.00	1.00	False	False	False	False	False	False	False	False	False	False	False	False	1.00	1.00
NZL	2	15	0.79	1.00	False	False	False	False	False	False	False	False	False	False	False	False	1.00	1.00
NZL	3	7	0.58	0.95	False	False	False	False	False	False	False	False	False	False	False	False	0.95	0.95
NZL	4	4	0.38	0.34	False	False	False	False	False	False	False	False	False	False	False	False	0.25	0.25
NZL	5	1	0.19	0.69	False	False	False	False	False	False	False	False	False	False	False	False	0.38	0.69

Table S17: Winning streak statistics for team New Zealand in *Test* format. In the table n denotes the length of the winning streak, f is the corresponding frequency of occurrence. $p(n)$ and $p(n_f)$ are the *p-values* for observation of streaks with length n and streaks with length n conditional on frequency f. With the help of multiple hypothesis tests mentioned in the previous section, we check the significance of the *p-values* and note down the results. We write result to be True, for a positive result, associated with a significant *p-value* below the standard confidence level of 0.05, otherwise it is False.

Country	n	f	$p(n)$	$p(n_f)$	Sidak		Holm		Hochberg		Bonferroni		Classic FDR		Storey FDR		Storey's Q	
.	.	.	.	.	$p(n)$	$p(n_f)$	$p(n)$	$p(n_f)$	$p(n)$	$p(n_f)$	$p(n)$	$p(n_f)$	$p(n)$	$p(n_f)$	$p(n)$	$p(n_f)$	$p(n)$	$p(n_f)$
PAK	1	28	1.00	1.00	False	False	False	False	False	False	False	False	False	False	False	False	1.00	1.00
PAK	2	10	0.85	1.00	False	False	False	False	False	False	False	False	False	False	False	False	1.00	1.00
PAK	3	9	0.69	0.95	False	False	False	False	False	False	False	False	False	False	False	False	0.95	0.95
PAK	4	7	0.54	0.38	False	False	False	False	False	False	False	False	False	False	False	False	0.21	0.21
PAK	5	3	0.39	0.60	False	False	False	False	False	False	False	False	False	False	False	False	0.08	0.08
PAK	6	2	0.25	0.49	False	False	False	False	False	False	False	False	False	False	False	False	0.20	0.20
PAK	8	1	0.08	0.33	False	False	False	False	False	False	False	False	False	False	**True**	**True**	0.02	0.05

Table S18: Winning streak statistics for team Pakistan in *Test* format. In the table n denotes the length of the winning streak, f is the corresponding frequency of occurrence. $p(n)$ and $p(n_f)$ are the *p-values* for observation of streaks with length n and streaks with length n conditional on frequency f. With the help of multiple hypothesis tests mentioned in the previous section, we check the significance of the *p-values* and note down the results. We write result to be True, for a positive result, associated with a significant *p-value* below the standard confidence level of 0.05, otherwise it is False.

Country	n	f	$p(n)$	$p(n_f)$	Sidak		Holm		Hochberg		Bonferroni		Classic FDR		Storey FDR		Storey's Q	
.	.	.	.	.	$p(n)$	$p(n_f)$	$p(n)$	$p(n_f)$	$p(n)$	$p(n_f)$	$p(n)$	$p(n_f)$	$p(n)$	$p(n_f)$	$p(n)$	$p(n_f)$	$p(n)$	$p(n_f)$
SAF	1	22	1.00	1.00	False	False	False	False	False	False	False	False	False	False	False	False	1.00	1.00
SAF	2	13	0.86	1.00	False	False	False	False	False	False	False	False	False	False	False	False	1.00	1.00
SAF	3	8	0.71	0.99	False	False	False	False	False	False	False	False	False	False	False	False	0.99	0.99
SAF	4	7	0.57	0.66	False	False	False	False	False	False	False	False	False	False	False	False	0.66	0.66
SAF	5	2	0.43	0.92	False	False	False	False	False	False	False	False	False	False	False	False	0.85	0.92
SAF	6	3	0.29	0.33	False	False	False	False	False	False	False	False	False	False	False	False	0.17	0.17
SAF	7	1	0.18	0.64	False	False	False	False	False	False	False	False	False	False	False	False	0.36	0.64
SAF	10	3	0.03	0.00	False	**True**	False	**True**	**True**	**True**	False	**True**	**True**	**True**	**True**	**True**	0.03	0.00

Table S19: Winning streak statistics for team South Africa in *Test* format. In the table n denotes the length of the winning streak, f is the corresponding frequency of occurrence. $p(n)$ and $p(n_f)$ are the *p-values* for observation of streaks with length n and streaks with length n conditional on frequency f. With the help of multiple hypothesis tests mentioned in the previous section, we check the significance of the *p-values* and note down the results. We write result to be True, for a positive result, associated with a significant *p-value* below the standard confidence level of 0.05, otherwise it is False.

Country	n	f	$p(n)$	$p(n_f)$	Sidak		Holm		Hochberg		Bonferroni		Classic FDR		Storey FDR		Storey's Q	
.	.	.	.	.	$p(n)$	$p(n_f)$	$p(n)$	$p(n_f)$	$p(n)$	$p(n_f)$	$p(n)$	$p(n_f)$	$p(n)$	$p(n_f)$	$p(n)$	$p(n_f)$	$p(n)$	$p(n_f)$
SRL	1	17	1.00	1.00	False	False	False	False	False	False	False	False	False	False	False	False	1.00	1.00
SRL	2	15	0.82	1.00	False	False	False	False	False	False	False	False	False	False	False	False	1.00	1.00
SRL	3	4	0.63	1.00	False	False	False	False	False	False	False	False	False	False	False	False	1.00	1.00
SRL	4	3	0.45	0.79	False	False	False	False	False	False	False	False	False	False	False	False	0.79	0.79
SRL	5	1	0.28	0.84	False	False	False	False	False	False	False	False	False	False	False	False	0.55	0.84
SRL	7	1	0.07	0.29	False	False	False	False	False	False	False	False	False	False	**True**	**True**	0.01	0.02
SRL	9	1	0.01	0.06	**True**	False	**True**	False	**True**	False	**True**	False	**True**	False	**True**	False	0.03	0.06

Table S20: Winning streak statistics for team Sri Lanka in *Test* format. In the table n denotes the length of the winning streak, f is the corresponding frequency of occurrence. $p(n)$ and $p(n_f)$ are the *p-values* for observation of streaks with length n and streaks with length n conditional on frequency f. With the help of multiple hypothesis tests mentioned in the previous section, we check the significance of the *p-values* and note down the results. We write result to be True, for a positive result, associated with a significant *p-value* below the standard confidence level of 0.05, otherwise it is False.

Country	n	f	$p(n)$	$p(n_f)$	Sidak		Holm		Hochberg		Bonferroni		Classic FDR		Storey FDR		Storey's Q	
.	.	.	.	.	$p(n)$	$p(n_f)$	$p(n)$	$p(n_f)$	$p(n)$	$p(n_f)$	$p(n)$	$p(n_f)$	$p(n)$	$p(n_f)$	$p(n)$	$p(n_f)$	$p(n)$	$p(n_f)$
WIN	1	34	1.00	1.00	False	False	False	False	False	False	False	False	False	False	False	False	1.00	1.00
WIN	2	16	0.85	1.00	False	False	False	False	False	False	False	False	False	False	False	False	1.00	1.00
WIN	3	12	0.69	1.00	False	False	False	False	False	False	False	False	False	False	False	False	1.00	1.00
WIN	4	4	0.54	0.98	False	False	False	False	False	False	False	False	False	False	False	False	0.98	0.98
WIN	5	1	0.38	0.99	False	False	False	False	False	False	False	False	False	False	False	False	0.76	0.99
WIN	6	2	0.24	0.49	False	False	False	False	False	False	False	False	False	False	False	False	0.17	0.17
WIN	7	2	0.13	0.15	False	False	False	False	False	False	False	False	False	False	False	False	0.15	0.15
WIN	8	1	0.07	0.29	False	False	False	False	False	False	False	False	False	False	**True**	**True**	0.01	0.02
WIN	17	1	0.00	0.00	**True**	**True**	**True**	**True**	**True**	**True**	**True**	**True**	**True**	**True**	**True**	**True**	0.00	0.00

Table S21: Winning streak statistics for team West Indies in *Test* format. In the table n denotes the length of the winning streak, f is the corresponding frequency of occurrence. $p(n)$ and $p(n_f)$ are the *p-values* for observation of streaks with length n and streaks with length n conditional on frequency f. With the help of multiple hypothesis tests mentioned in the previous section, we check the significance of the *p-values* and note down the results. We write result to be True, for a positive result, associated with a significant *p-value* below the standard confidence level of 0.05, otherwise it is False.

Country	n	f	$p(n)$	$p(n_f)$	Sidak		Holm		Hochberg		Bonferroni		Classic FDR		Storey FDR		Storey's Q	
.	.	.	.	.	$p(n)$	$p(n_f)$	$p(n)$	$p(n_f)$	$p(n)$	$p(n_f)$	$p(n)$	$p(n_f)$	$p(n)$	$p(n_f)$	$p(n)$	$p(n_f)$	$p(n)$	$p(n_f)$
ZIM	1	8	1.00	1.0	False	False	False	False	False	False	False	False	False	False	False	False	1.00	1.0
ZIM	2	2	0.49	1.0	False	False	False	False	False	False	False	False	False	False	False	False	0.99	1.0

Table S22: Winning streak statistics for team Zimbabwe in *Test* format. In the table n denotes the length of the winning streak, f is the corresponding frequency of occurrence. $p(n)$ and $p(n_f)$ are the *p-values* for observation of streaks with length n and streaks with length n conditional on frequency f. With the help of multiple hypothesis tests mentioned in the previous section, we check the significance of the *p-values* and note down the results. We write result to be True, for a positive result, associated with a significant *p-value* below the standard confidence level of 0.05, otherwise it is False.

References

[1] Yoav Benjamini and Yosef Hochberg. Controlling the false discovery rate: a practical and powerful approach to multiple testing. *Journal of the Royal statistical society: series B (Methodological)*, 57(1):289–300, 1995.

[2] Giovanni Fasano and Alberto Franceschini. A multidimensional version of the kolmogorov–smirnov test. *Monthly Notices of the Royal Astronomical Society*, 225(1):155–170, 1987.

[3] Yosef Hochberg. A sharper bonferroni procedure for multiple tests of significance. *Biometrika*, 75(4):800–802, 1988.

[4] Sture Holm. A simple sequentially rejective multiple test procedure. *Scandinavian journal of statistics*, pages 65–70, 1979.

[5] John A Peacock. Two-dimensional goodness-of-fit testing in astronomy. *Monthly Notices of the Royal Astronomical Society*, 202(3):615–627, 1983.

[6] Tuomas Puoliväli, Satu Palva, and J Matias Palva. Influence of multiple hypothesis testing on reproducibility in neuroimaging research: A simulation study and python-based software. *Journal of Neuroscience Methods*, page 108654, 2020.

[7] Zbyněk Šidák. Rectangular confidence regions for the means of multivariate normal distributions. *Journal of the American Statistical Association*, 62(318):626–633, 1967.

[8] John D Storey and Robert Tibshirani. Statistical significance for genomewide studies. *Proceedings of the National Academy of Sciences*, 100(16):9440–9445, 2003.

[9] J Timmer and M Koenig. On generating power law noise. *Astronomy and Astrophysics*, 300:707, 1995.
